# Keystone Epitope Theory: An Ecological Perspective on RNA Viruses, Tumor Immunoediting, and Vaccine Design

**DOI:** 10.20411/pai.v11i2.1011

**Published:** 2026-08-07

**Authors:** Simon Mallal, Amir Asiaee

**Affiliations:** 1 Department of Medicine, Vanderbilt University Medical Center, Nashville, Tennessee; 2 Institute for Immunology and Infectious Diseases, Murdoch University, Perth, Western Australia; 3 Department of Biostatistics, Vanderbilt University Medical Center, Nashville, Tennessee

**Keywords:** Immunodominance, Peptide–HLA, TCR, KIR, NKG2A, Decoy Epitope, Antigen Display Control, SARS-CoV-2, HIV, Tumor Immunoediting, Vaccine Design, Keystone Epitope Theory

## Abstract

We propose that persistent, human-adapted DNA organisms shape postnatal immunity by focusing responses on functionally constrained epitopes within tissue niches. Here, we examine rapidly evolving RNA viruses and tumors through that lens. We propose that their persistence is promoted by 2 coupled mechanisms: (i) immunodominance steering toward mutable “decoy” epitopes that contribute little to durable control, and (ii) antigen display control that reduces cytotoxic T lymphocyte (CTL) recognition while preserving inhibitory natural killer (NK) receptor engagement, for example via HIV Nef/Vpu effects on HLA-A and HLA-B and through HLA-E/NKG2A pathways. Tumors show analogous vulnerabilities through altered class I expression and reinforcement of inhibitory signaling. Using HIV as the primary model, we distinguish HLA-associated viral adaptation mechanisms and highlight evidence consistent with a subset of adaptations that preserve detectable T-cell recognition while being associated with reduced antiviral effector function. We then consider the degree to which this framework can be extended to hepatitis C virus (HCV), influenza, SARS-CoV-2, and tumor immunoediting. We conclude with 3 vaccine design principles: prioritize epitopes where substitutions carry measurable fitness costs, avoid immunogens dominated by mutable targets, and account for antigen presentation context and inhibitory NK signaling when evaluating epitope choice. We distinguish established observations from testable predictions and outline experiments needed to evaluate the framework. We frame the analysis conditionally on the keystone-imprinting premise, which is developed in companion work.

## KEYSTONE EPITOPE THEORY IN BRIEF

**Figure 1.**
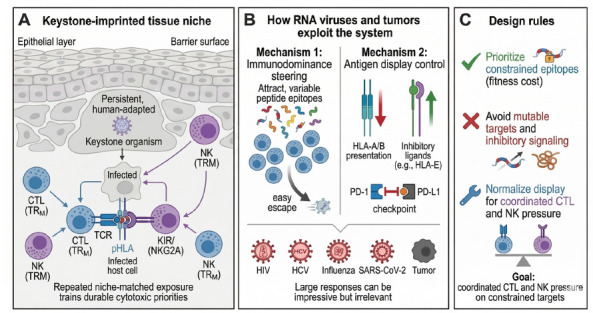
**Keystone Epitope Theory at a glance.** Keystone organisms (persistent, human-adapted infections) repeatedly restimulate constrained pHLA targets in tissue niches, installing durable immune priorities that coordinate CD8, CD4, B-cell, and NK programs. Persistence of fast-evolving RNA viruses is associated with inflating mutable decoys (immunodominance steering) and altering antigen display, thereby reducing CTL visibility while preserving or rerouting inhibitory NK signaling. Tumors converge on the same vulnerability via class-I/E rewiring and checkpoint reinforcement. Practical implication: prioritize constrained epitopes with measurable fitness cost, avoid decoy-heavy designs, and normalize display when required so cytotoxic gains are visible to both CTL and NK arms.

We propose that a small group of persistent, human-adapted infections—notably herpesviruses such as cytomegalovirus (CMV), Epstein-Barr virus (EBV), human herpesvirus 6 (HHV-6), varicella-zoster virus (VZV) and herpes simplex virus type 1 (HSV-1) and -2 and *Mycobacterium tuberculosis*—may disproportionately shape postnatal immune priorities by repeatedly expressing epitopes coordinating functionally linked immune responses in the tissue niches where control must occur [1]. The term “keystone” borrows from ecology, where a keystone species maintains ecosystem structure beyond its abundance alone. In this framework, keystone organisms that have co-evolved with humans over long co-evolutionary time anchor durable protective immune memory in specific niches to the benefit of both host and persistent organism. In contrast, mutable decoy epitope*s* within highly adaptable organisms may exploit this architecture to elicit robust but non-protective responses (Figure 1). A single peptide–HLA (pHLA) complex on the cell surface is read by 2 cytotoxic arms: CD8 T cells via the T-cell receptor (TCR) and natural killer (NK) cells via KIR and NKG2 receptors. Because CTLs and NK cells interrogate overlapping HLA class I surfaces with different receptor logic, successful escape depends on uncoupling CTL loss from NK rescue—not on simple HLA downregulation alone. Pathogens and tumors can achieve this by selectively remodeling class I and associated ligands so that CTL-relevant pHLA falls while inhibitory NK signaling is preserved or rerouted [2].

The evidence that keystone organisms imprint postnatal immune hierarchies in this way is developed in companion work and is, at present, a hypothesis rather than an established mechanism [1–3]. We therefore frame this review conditionally: we ask what follows for rapidly evolving RNA viruses and tumors if keystone imprinting operates as proposed. Where we summarize supporting observations, we do so to make the review self-contained, not to imply that the premise is settled. Briefly, several observations motivate it: (i) a small set of persistent, human-adapted organisms (notably CMV, EBV, VZV, HSV-1 and -2, HHV-6, and *M. tuberculosis*) account for a substantial fraction of tissue-resident and circulating memory CD8 T cells in adults [4]; (ii) latent carriage of some of these organisms can raise basal immune readiness and confer heterologous protection in animal models [5, 6]; and (iii) a trait-based analysis identifies features that distinguish these organisms from transient or environmental exposures [1]. These observations are consistent with, but do not by themselves establish, the proposed imprinting mechanism.

We also propose a candidate compartment-level summary metric: the Keystone Reactivation Index, defined for a sampled tissue compartment as the number of keystone-organism detections divided by the total number of microbial detections in that compartment (for example, from metagenomic sequencing or multiplex PCR of a tissue or fluid sample; Supplementary Material S7.1) [1, 7]. A rising Keystone Reactivation Index would mean that keystone organisms make up a growing share of the local microbial signal, which we hypothesize reflects disruption of keystone equilibrium with-in that compartment. If prospectively validated, the Keystone Reactivation Index could serve as a candidate compartment-level summary metric for tracking immune perturbation and for identifying settings in which decoy-dominant misallocation is most likely. Neither application has yet been prospectively tested. Three considerations follow from this framework that extend standard escape and immunodominance models: (i) CTL and NK arms interrogate overlapping HLA class I surfaces with different receptor logic, so successful escape depends on uncoupling CTL loss from NK rescue rather than on simple HLA downregulation alone; pathogens and tumors can do this by selectively remodeling class I and associated ligands so CTL-relevant pHLA falls while inhibitory NK signaling is preserved or rerouted [2]—a vulnerability not captured by models that treat T-cell escape alone; (ii) the protective value of an epitope depends on tissue compartment and exposure history, not only on sequence conservation or response magnitude; and (iii) a subset of HLA-associated viral substitutions may retain T-cell recognition while shifting effector function toward non-protective states, a testable prediction distinguishable from conventional exhaustion or escape.

Here, we focus on RNA viruses and tumors as ecological disruptors of keystone-imprinted hierarchies, and on the vaccine design principles that follow from this analysis. Our aim is to keep the design implications high-level: what to target, what to avoid, and when to normalize display so immunogens and therapies work with, rather than against, entrenched immune priorities. A full glossary of key terms is provided in Supplementary Material S1. For a comprehensive primer with section pointers, see Supplementary Material S2.1. For historical context and reflections on the conceptual impact of MHC restriction, see [8].

Throughout this review, the term “*adaptation*” refers to reproducible HLA-associated viral polymorphisms in the population-genetic sense [9, 10]; we specify mechanism only where it has been established.

## RNA VIRUSES AS DISRUPTORS OF KEYSTONE HIERARCHIES

**Figure 2.**
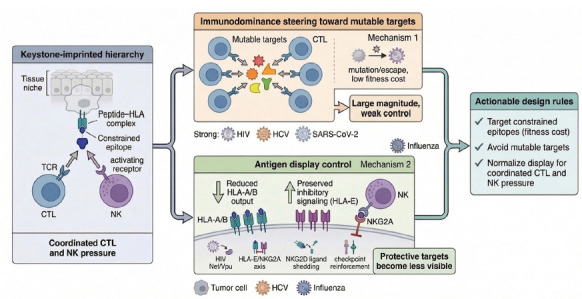
**Two coupled mechanisms of RNA-virus and tumor subversion.** The schematic maps HIV, HCV, influenza, and SARS-CoV-2 examples to actionable design rules, organized by immunodominance steering and antigen display control.

In this framework, persistent human-specific co-evolved organisms that focus immunity on epitopes that elicit coordinated dynamic immune responses in specific niches give humans speed and reliability against other threats. RNA viruses and tumors may succeed when they successfully negotiate and exploit this landscape: inflating mutable decoys and altering display so cytotoxic T-cell visibility falls while inhibitory NK signaling is preserved or rerouted. Fast-evolving RNA viruses and tumor immunoediting provide particularly informative tests of this framework. HIV continuously probes what is presented on pHLA surfaces and how that visibility is interpreted by CTL and NK circuits, revealing where immune hierarchies may be brittle [11]. Tumors run the same search in miniature, evolving toward antigen loss, altered class I/E display, and checkpoint-mediated immune evasion [12–14]. If persistent DNA viruses shape immune priorities within tissue niches as proposed, then rapidly evolving RNA viruses and tumors provide a stringent test because they persist in hosts whose immune hierarchies are already established. HIV is particularly informative because its rapid within-host evolution allows residue-level associations between host HLA alleles and viral sequence variation to be measured, providing an empirical readout of HLA-restricted selection pressures [9, 15]. We propose 2 broad routes by which RNA viruses persist (Figure 2): (i) preferential amplification of responses directed toward mutable epitopes that contribute little to durable control, and (ii) changes in cell-surface pHLA presentation that reduce CTL recognition while maintaining ligands that engage inhibitory NK receptors. Tumors can converge on the same vulnerabilities through altered class I expression, increased display of inhibitory ligands, and reinforcement of checkpoint pathways.

**Figure 3.**
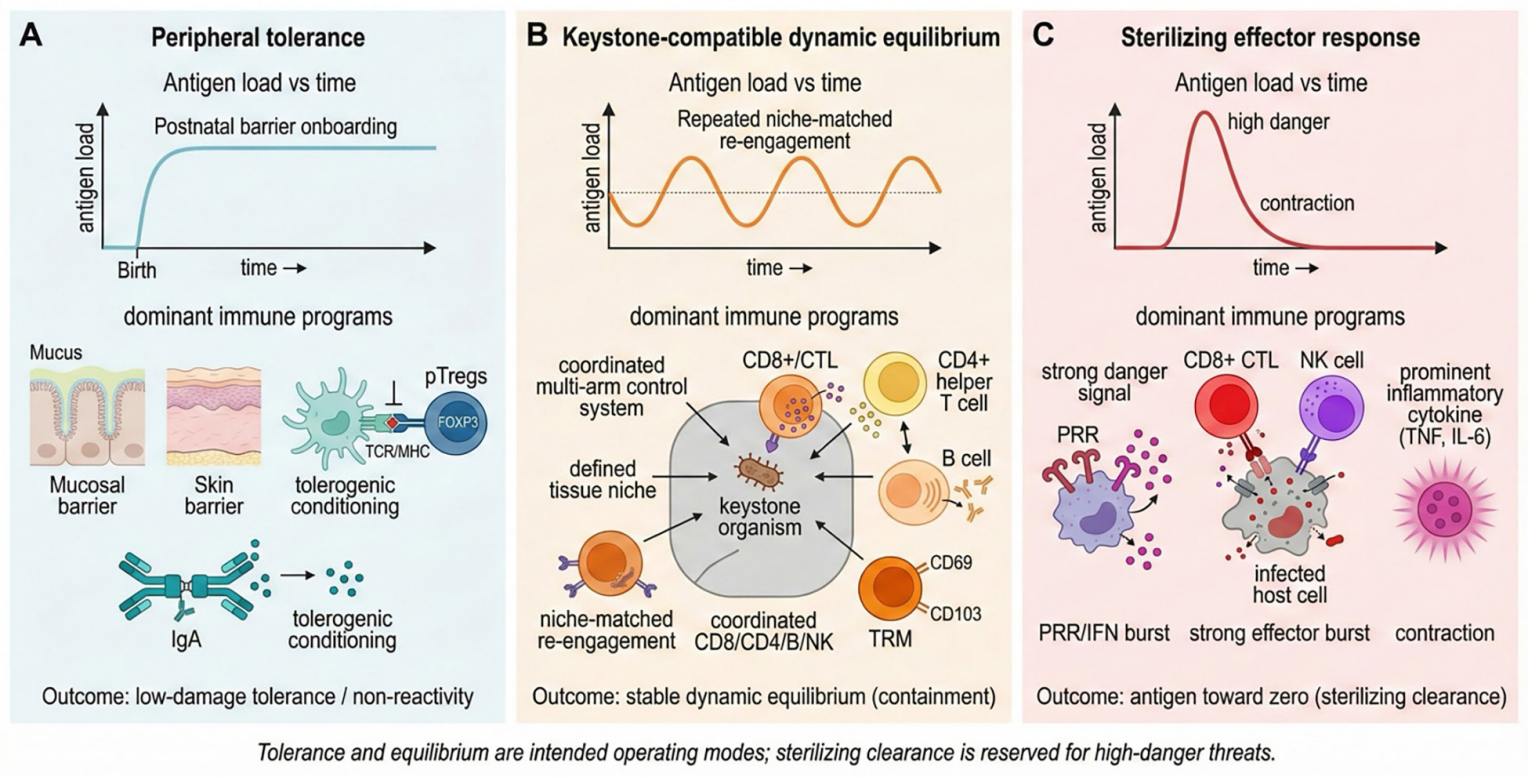
**Three immune operating modes across the antigenic landscape: tolerance, equilibrium, and sterilization.** (A) Tolerance predominates for high-abundance, low-danger exposures, especially after birth at mucosal and skin surfaces, supported by pTregs, tolerogenic antigen-presenting cell conditioning, and IgA-based immune exclusion. (B) Equilibrium maintains long-term containment of co-evolved residents through coordinated CD8/CD4/B/NK programs with tissue-resident memory. (C) Sterilization drives antigen toward 0 in high-danger acute infection, then contracts. Tolerance and equilibrium are intended operating modes, not failures.

We propose that immune outcomes are better understood as qualitatively distinct operating modes than as points on a single continuum of response strength. Throughout, we use three terms consistently: tolerance, equilibrium, and sterilization (Figure 3). Tolerance refers to peripheral tolerance and non-inflammatory barrier control in response to high-abundance, low-risk exposures. Equilibrium refers to stable, compartmentalized containment of co-evolved residents through coordinated CD8/CD4/B/NK programs and tissue-resident memory, without sterilizing the organism and without chronic immunopathology. Sterilization refers to high-intensity effector programs that eliminate the last vestiges of infection in high-danger acute infection and then contract. RNA viruses and tumors can shift responses away from equilibrium, either toward misallocated tolerance or toward high-magnitude but strategically irrelevant sterilization attempts. In this framing, tolerance in barrier tissues is often not an absence of immunity but a thresholded control program that retains competent effectors while regulating output through local inhibitory regulation. This matters for RNA viruses because immunopathology frequently reflects forced threshold breach rather than de novo autoimmunity, especially when inflammatory gain is pharmacologically or virally amplified [16].

We organize the mechanisms by which RNA viruses and tumors subvert immunity as 2 coupled mechanisms: (i) immunodominance steering, the preferential expansion of responses toward non-protective or mutable decoy targets; and (ii) antigen display control, the selective alteration of cell-surface pHLA presentation that reduces CTL visibility while preserving or rerouting inhibitory NK signaling [2]. This organizing framework is summarized in Figure 2.

**Figure 4.**
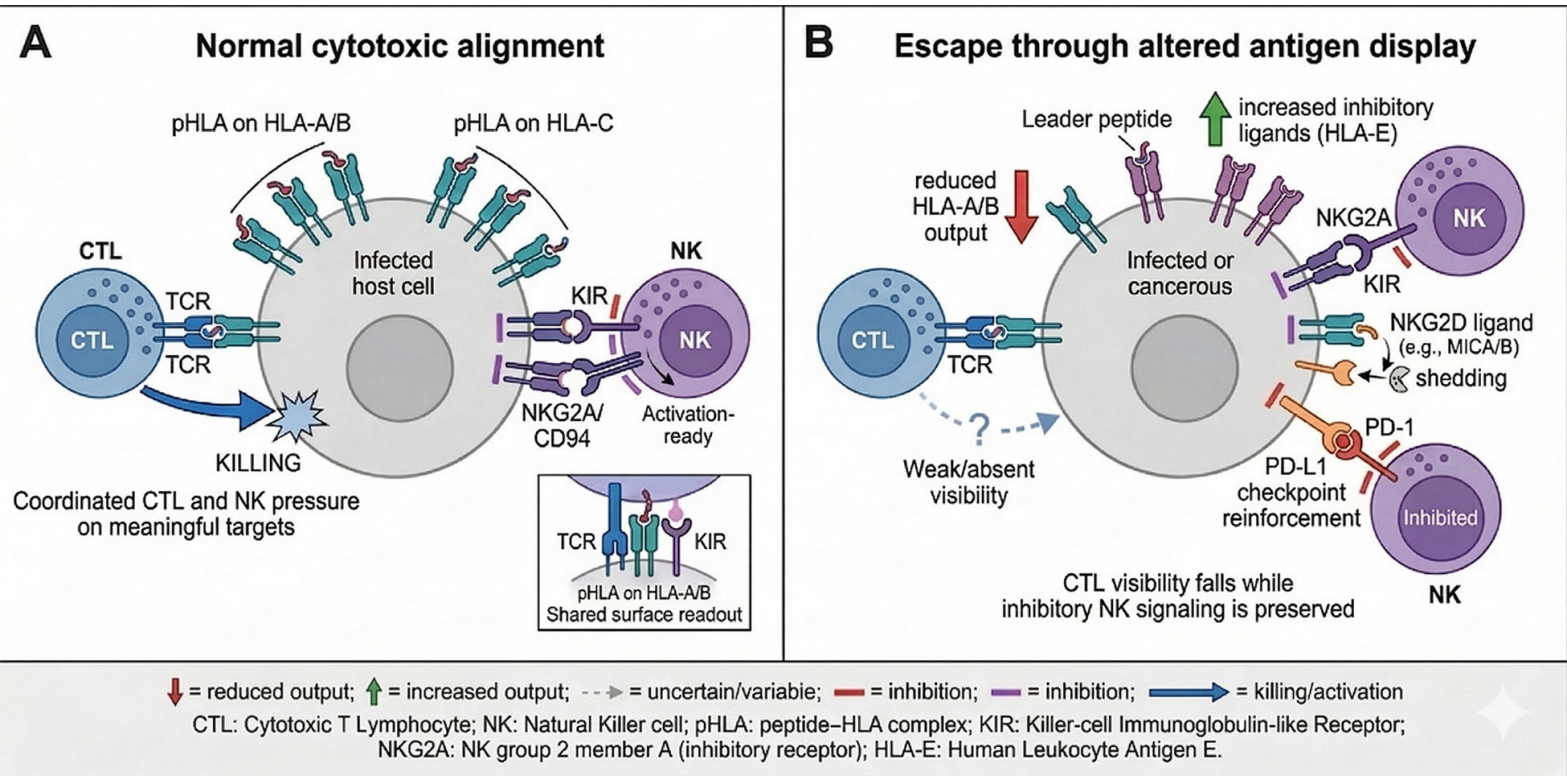
**The shared CTL–NK vulnerability.** Pathogens and tumors converge on antigen-display strategies that can weaken CTL pressure without triggering NK rescue: selective reduction of HLA-A/B output, host- and strain-dependent tuning of HLA-C, preservation of HLA-E/NKG2A signaling, peptide-dependent KIR effects, and checkpoint reinforcement. Simple class I loss can activate NK cells; durable escape generally requires these additional layers of selective remodeling [2].

The shared CTL–NK vulnerability—the problem of reducing CTL-relevant pHLA without triggering effective NK rescue, through selective remodeling of class I ligands and checkpoints—is schematized in Figure 4 [2].

**Figure 5.**
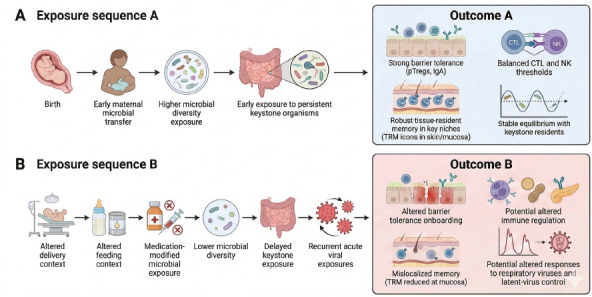
**Hypothesis: How variation in early-life exposure timing may influence immune calibration.** Upper panel: early sequential exposure in ancestral life to persistent infections (Exposure sequence A). Lower panel: altered timing and order of modern-day exposures (Exposure sequence B). The hypothesis is that differences in timing and sequence—not the presence or absence of any single intervention—may shift immune priorities. Important: This figure does not argue that modern medical interventions are harmful. Vaccination, antibiotics, and sanitation are overwhelmingly beneficial. The hypothesis is that the timing and sequence of certain antigenic exposures may influence immune calibration, and that understanding this could improve vaccine design. Vaccines such as those for VZV and YFV already achieve keystone-like imprinting without the morbidity of natural infection [20, 21] (Supplementary Material S2.2).

Figure 5 illustrates how variation in the timing and sequence of early-life antigenic exposures could, in principle, alter subsequent immune calibration. The upper panel shows one hypothesized early exposure sequence typical of ancestral life; the lower panel shows how modern life alters timing and nature of antigen exposures to shift immune priorities.

Where early-life exposure to persistent keystone organisms is delayed or reordered, we predict that subsequent immune hierarchies may be calibrated differently—potentially altering how later RNA-virus challenges are handled [17]. This is a hypothesis about exposure timing and sequence, not an argument against any intervention. The early-life set of antigenic exposures that shapes immune priorities can differ by geography and context, and these differences may influence susceptibility to immunodominance steering by RNA viruses. If this hypothesis is correct, longitudinal tracking of the Keystone Reactivation Index could, in principle, serve as a candidate systems-level readout of calibration mismatch; however, this application remains unvalidated and would require prospective clinical studies to determine whether Keystone Reactivation Index shifts predict meaningful immunological outcomes [1, 17].

HLA-B diversification may in part reflect selective pressure from RNA viruses, as more recently diverged HLA-B alleles show enhanced capacity to present RNA-virus-derived epitopes and HLA-B targeting efficiency correlates with HIV and dengue outcomes [18, 19], though any direct causal relationship cannot be established.

## DECOY VS CONSTRAINED EPITOPES

A practical challenge in HIV immunogen design is distinguishing epitopes where HLA-associated viral variation is limited by fitness costs from those that are strongly immunogenic but do not support durable control. Sequence conservation describes a pattern in an alignment; constraint is a mechanistic property—the demonstration that substitutions at a given site carry a measurable fitness cost—and must be established experimentally. Constrained epitopes are those in which substitutions measurably impair replication capacity, protein folding, or catalytic function [22–24]. The same principle is familiar from antiretroviral therapy: drugs targeting catalytically indispensable residues face resistance pathways that carry substantial fitness penalties. Sequence conservation is often used as a surrogate for constraint, but mutagenesis studies and direct fitness measurements are more specific than conservation [22, 23]; constraint is best treated as an empirical viral fitness property, not inferred from conservation. An important caveat is that mutational constraint is not one-dimensional. HIV adaptation pathways can become epistatically entrenched: fitness-costly HLA-associated substitutions may persist after transmission because compensatory or precursor mutations partially restore replication and delay reversion, while alternative routes with different costs can emerge over time [25, 26].

The selective value of any candidate target also varies with viral genetic background and host population: the same HLA allele can have different clinical associations across clades, the same clade can show different adaptation pathways in different populations, and subtype-specific viral context can alter both peptide–HLA binding and the fitness cost of adaptation [27–29]. Population-level HIV adaptation to local HLA distributions and the transmission of preadapted virus further indicate that conservation or single-mutation fitness costs alone cannot define universal vaccine targets [10, 30–32]. Accordingly, candidate epitopes should be prioritized not only for measurable constraint but also for escape-route limitation, low propensity for compensatory entrenchment, favorable clinical associations across populations, and reproducible function under natural processing and presentation.

We define decoy epitopes as targets that elicit robust, often immunodominant, responses yet contribute little to sustained virological control, commonly because substitutions at these sites are well tolerated. In HIV, cohort and cellular data support disproportionate expansion of Nef-directed CD8 responses in some settings, with relative underrepresentation of responses to Gag regions more consistently associated with lower viral load [33, 34]. The term “decoy” refers to this functional pattern at the level of immune allocation and virologic outcome; it does not require assumptions about viral intent.

To keep the term operational, we distinguish a Keystone Epitope Theory (KET)-specific decoy from an epitope that is simply immunodominant and ineffective for independent reasons. An epitope qualifies as a KET-specific decoy only if (i) its dominance is not fully explained by intrinsic peptide–HLA properties (binding affinity, precursor frequency, processing efficiency); (ii) its dominance covaries with keystone exposure history, age, or geography in a way that intrinsic immunogenicity alone does not predict; and (iii) redirecting responses away from it measurably improves control. In the absence of (ii) and (iii), the functional decoy label should be applied descriptively, without the further claim that keystone imprinting steers the response. Current HIV data establish the functional decoy pattern for Nef-directed responses and bear on criterion (iii), but do not yet test criterion (ii); we therefore treat Nef epitopes as candidates, rather than confirmed, KET-specific decoys.

Even when decoy epitopes are simply ineffective in the neutral sense, their inclusion in immunogens can be disadvantageous because immunodominance is competitive: immune resources are finite, and responses directed toward one set of targets necessarily reduce the capacity to respond to others. Constraints can also exist at the RNA layer, including codon usage and RNA structural constraints that influence mutational accessibility and viral evolution [35, 36], which can matter when epitope selection relies heavily on sequence-based criteria.

Conserved-region vaccine programs, notably the HIVconsv and second-generation HIVconsvX platforms, represent the most direct experimental tests of the premise that redirecting T-cell responses away from variable immunodominant targets can improve outcomes [37, 38]. HIVconsvX goes beyond crude sequence conservation: its design combines functionally conserved Gag and Pol regions with bivalent complementary mosaics and enrichment for epitopes associated with lower viral loads in untreated infection [38].

In phase 1 studies, HIVconsvX elicited broad, polyfunctional CD8 T-cell responses capable of *in vitro* inhibition of HIV-1 clades A through D [39, 40]. These results confirm that immune refocusing toward conserved targets is feasible and immunogenic. However, they do not yet resolve a deeper ambiguity: amino-acid conservation can arise from heterogeneous causes, including protein-level fitness constraints, RNA structural requirements, historical HLA-driven adaptation at the population level, and context-dependent antigen processing or display. Consistent with this, Rolland et al engineered 23 single amino-acid substitutions in a subtype B p24 center-of-tree backbone and measured replicative fitness by direct competition assay [30].

Although some mutations at highly conserved sites were lethal or costly, there was no strong overall relationship between the database frequency of the consensus residue (the metric used to define conservation) and replicative capacity. Sites that had changed in frequency between the 1980s and 2000s circulating sequences were enriched for HLA-associated positions and tended to carry only modest fitness costs, whereas temporally stable sites carried greater costs. The authors concluded that vaccine design should prioritize sites that remain conserved despite immune-selective pressure, not merely sites that appear conserved in a cross-sectional alignment [30]. Notably, the HIVconsvX designers themselves acted on related logic by excluding several highly conserved regions that contained only epitopes associated with unfavorable clinical outcomes [38], a direct acknowledgment from within the field that conservation alone is not a sufficient selection criterion. Moreover, 4 of 6 HIVconsvX regions derive from Pol, a gene product expressed at roughly 1/20th the abundance of Gag owing to the ribosomal frameshift mechanism, raising the possibility that some conserved Pol epitopes are poorly displayed on infected cells despite their sequence-level constraint [38]. The critical design problem is therefore not simply conserved vs variable, but distinguishing conserved targets that are both virologically constrained and immunologically actionable from those that are conserved yet clinically non-productive *in vivo*.

A narrower possibility is also worth considering: beyond residues conserved because substitutions are structurally or catalytically costly, some near-invariant targets may persist for reasons not fully captured by protein-level fitness cost alone, including stable engagement of pre-existing immune hierarchies shaped by prior exposures. This remains speculative and would require direct experimental testing. HIV-1 group M entered the human population carrying ancestral primate lentiviral immune-evasion mechanisms and has since been adapting to host-population HLA profiles over approximately a century of transmission [10, 31]; within this timeframe, conservation at some positions may reflect immune co-evolutionary stability rather than functional constraint. This mechanism, if operative, would constitute a second stratum of decoy activity invisible to amino-acid frequency analysis and to current conserved-element selection algorithms. It remains a hypothesis rather than an established mechanism, but it is consistent with the Rolland et al observation that conservation and fitness cost are not synonymous [30], and it generates specific experimental predictions outlined in Supplementary Material S6.

We frame decoy susceptibility as a consequence of keystone-imprinted prioritization, in which fast-evolving RNA viruses restimulate high-salience memory that is poorly protective against viral targets [1]. A practical extension is that keystones are expected to dominate species-wide priors, but compartmental priors may also arise from specialist niche residents and recurrent environmental exposures [41]. Therefore, decoy susceptibility may vary with geography, age, and exposure history, consistent with mismatch effects described for vaccine take and regional calibration. A separate possibility, that a subset of adaptations preserves detectable recognition while shifting effector function in a direction that favors persistence, is discussed below.

## ANTIGEN DISPLAY AND THRESHOLD CONTROL

HIV functions as a stringent test of this framework because it simultaneously undermines adaptive recognition at 2 levels. At the epitope level, rapid mutation inflates immunodominant responses toward mutationally permissive decoy sites; at the cell-surface level, Nef and Vpu reshape class I display to preserve inhibitory NK signaling, including NKG2A/KIR pathways, while eroding CTL visibility [2, 42]. Among RNA viruses, HIV is a particularly informative example of immune subversion through rapid evolution. Produced at over 2 billion virions daily, driven by error-prone reverse transcriptase and high recombination [43, 44], HIV treats each amino acid position as a quasi-independent evolutionary outcome. This extreme adaptability makes HIV particularly informative: its capacity to respond at the residue-specific level provides an empirical readout of the selective pressures attributed to keystone imprinting, highlighting which sites are under consistent immune selection and how immunodominant hierarchies are shaped and subverted. At the population level, this adaptation manifests as consistent associations between specific HLA alleles and viral amino acid polymorphisms, a pattern first demonstrated in a population study of HIV adaptation to HLA-restricted responses [9].

HIV provides a concrete case of how immunodominance often goes “wrong” in RNA infections. Cohort and cellular data support disproportionate expansion of Nef-directed CD8 responses in some settings, at the expense of more protective Gag-specific responses [34]. Deep sequencing studies show that mutations within immunodominant epitopes often persist despite immune pressure because they impose little fitness cost [45]. Codon usage analysis suggests that HIV is pre-adapted to rapidly adapt to HLA allele-specific selective pressure [35]. Some of these substitutions may preserve immunogenicity while reducing the effectiveness of the resulting CD8+ T-cell response, though this interpretation requires functional validation for each epitope [34, 46–48]. At a population level, HIV mutations within CTL epitopes are consistently associated with specific HLA alleles, suggesting that viral adaptation is actively shaped by host immune pressure [9]. However, such associations do not immediately distinguish between recognition-reducing adaptation (classical escape), in which selection eliminates immune recognition, and decoying, in which immune pressure is redirected toward non-protective targets; the latter requires further functional validation [45, 48, 49].

Population-level studies show that HIV evolves differently in distinct populations, with unique HLA-adapted polymorphisms emerging in genetically distinct host groups [28]. Transmission of pre-adapted HIV variants weakens subsequent immune responses, leading to higher viral loads and faster CD4+ T-cell decline [31], while epistatic interactions with host genetic factors such as ERAP2 further modulate adaptation and disease outcomes [50]. HLA-B57-associated substitutions rapidly revert when transmitted to non-B57 hosts, while other HLA-associated mutations remain stable, suggesting compensatory networks that lock in some adaptations [47]. The accumulation of HLA-associated substitutions associated with previously protective alleles such as HLA-B57 and HLA-B27 has led to a decline in their ability to mediate immune control [10]. Extended evidence for these HIV adaptation dynamics, including subsections on population-level viral adaptation, reversion dynamics, and implications for vaccine and drug resistance, is provided in Supplementary Material S3.

## RECOGNITION-PRESERVING ADAPTATION WITH REDUCED ANTIVIRAL FUNCTION

The preceding sections established that HLA-associated viral adaptation can reduce recognition of presented peptides, sometimes with measurable fitness costs [25, 51, 52], and that this well-supported framework informs immunogen strategies emphasizing fitness-constrained regions. Here we consider a narrower, testable extension: whether some adaptations retain detectable T-cell recognition while being associated with CD8 responses that show reduced antiviral effectiveness—a pattern distinct from classical escape.

The available evidence supports biological plausibility for recognition-preserving adaptations, while the prevalence of this phenomenon across epitopes and its net effect on viral fitness *in vivo* remain to be established. First, longitudinal sampling shows that immune selection can be followed by the emergence of variant-targeted responses with strong cytokine readouts and high avidity, without durable suppression of replication [49]. This observation is consistent with the general point that response magnitude and interferon (IFN)-γ production alone can be insufficient predictors of control. Second, single-cell and functional analyses support a mechanistic basis for recognition-preserving changes with altered function: a single amino acid substitution within a targeted epitope can preserve CD8 activation while shifting transcriptional programs and functional outputs, including reduced polyfunctionality and impaired cytotoxic activity [48]. These data do not, by themselves, prove a net fitness advantage to the virus, but they do support the plausibility of a phenotype in which an epitope remains immunogenic while becoming less effective for clearance. Third, population-level analyses provide a quantitative framework for testing net effects: where specific HLA-associated substitutions carry measurable fitness costs, these costs are reflected in patterns of reversion and in lower viral load among hosts with non-adapted residues compared to the population mean [10, 25, 31]. The same population-level analytic frame-work can be extended to test whether a subset of adaptations shows a different pattern: higher viral loads of those with adapted residues compared to the population mean and worse control specifically in HLA-matched hosts who retain T-cell recognition of the adapted epitope—a pattern that, if confirmed, would be consistent with recognition-preserving adaptation that benefits the virus.

An alternative explanation is that these patterns reflect partial recognition reduction coupled with chronic-stimulation-driven functional impairment, rather than a distinct adaptation class. Distinguishing these mechanisms requires paired comparisons of wild-type and adapted epitope forms within individuals, using direct cytotoxicity measurements and single-cell state profiling. For vaccine design, 2 implications follow: (i) immunogens can exclude highly variable immunodominant targets on conservative grounds, and (ii) if recognition-preserving but functionally ineffective adaptations are confirmed at scale, excluding the relevant epitopes becomes more important, as priming or restimulating such responses could be counterproductive. Consistent with these considerations, vaccine-elicited helper responses can be attenuated when epitopes are already HLA class II adapted in circulating virus [53].

On current evidence, recognition-preserving adaptation is documented for only a small number of well-characterized epitopes [48, 49], and the majority of characterized HLA-associated substitutions remain consistent with classical recognition-reducing adaptation. Whether recognition-preserving adaptation is confined to a few epitopes or is more widespread is itself unresolved. The population-level test described above (higher viral loads and worse control in HLA-matched hosts who retain recognition of the adapted epitope) is one way to estimate its prevalence.

## HEPATITIS C VIRUS

Hepatitis C virus (HCV) follows a distinct evolutionary trajectory from HIV. Rather than continuously reshaping its immunodominance profile through rapid HLA-associated sequence turnover, HCV exhibits constrained evolution where certain genotypes favor immune-associated variation while others maintain greater epitope conservation [54]. A comparative study of HCV genotypes 1 and 3 demonstrated divergent adaptation strategies, with genotype 1 showing more frequent HLA-associated variation and genotype 3 displaying greater conservation at immune-restricted sites [54]. Unlike HIV, HLA-associated substitutions in HCV tend to persist even in hosts lacking the relevant HLA allele, implying a fitness advantage [55]. Studies have also shown that HCV-specific CD8+ T cells from individuals with multiple exposures to different HCV genotypes maintain cross-reactivity, suggesting that immune imprinting against prior exposures can influence the ability to clear subsequent infections [56]. HCV also undergoes CD4+ T-cell-driven immune adaptation, with HLA class II-associated polymorphisms emerging in chronic infection [57], consistent with the possibility that prior immune architecture could shape both cytotoxic and helper T-cell response landscapes. Host genetics, particularly IL28B, HLA-C, and KIR variants, play a critical role in determining clearance outcomes [58], consistent with the possibility that keystone-driven immune responses coordinate both adaptive and innate immunity. HCV thus illustrates that the relative contributions of viral constraint, host genetics, and prior immune imprinting differ across RNA viruses. For vaccine design, HCV serves as a constraint-and-adaptation calibration case: Emphasis should fall on epitopes where adaptation is measurably costly, and “impressive responses” should be interpreted through the lens of whether they track with clearance rather than dominance alone.

## INFLUENZA

In influenza A (H1N1) infection, the efficiency with which HLA alleles target conserved viral epitopes correlates with CD8+ T-cell response magnitude and clinical outcomes [59]. HLA alleles that preferentially bind structurally constrained influenza epitopes are associated with stronger T-cell responses and lower mortality [59]. Conversely, HLA-A*24:02, which is common in several Indigenous populations, has been linked to severe influenza outcomes and a distinctive CD8 T-cell landscape [60]. Whether this reflects an active viral decoying strategy or simply the binding preferences of individual HLA alleles remains an open question; for vaccine design, the relevant variable is the functional outcome—misdirected immunodominance toward mutable targets, regardless of whether the mechanism is pathogen-driven or host-determined. Despite undergoing antigenic drift and shift, influenza retains conserved epitopes that function in a keystone-like manner, shaping protective immunodominance hierarchies. However, unlike keystone DNA viruses, which maintain long-term immune imprinting through persistent exposure, influenza-driven imprinting is transient and strain-dependent. Thus, influenza highlights that even among RNA viruses, a limited repertoire of epitopes is available because epitope selection is constrained by the structural and functional requirements of key proteins. We predict that protection tracks with efficient targeting of conserved epitopes rather than response magnitude alone; candidates should be evaluated by whether they improve functional outcomes.

## SARS-COV-2

SARS-CoV-2 provides a distinct test case. Unlike highly mutable viruses such as HIV and HCV, SARS-CoV-2 operates within a more stable immunological framework, engaging pre-existing immune hierarchies shaped by prior coronavirus or herpesvirus exposure rather than continuously evading immune memory [61]. Studies demonstrate that 20% to 50% of unexposed individuals have detectable T-cell reactivity against SARS-CoV-2, largely driven by cross-reactive memory from common cold coronaviruses [61, 62]. At the level of innate-adaptive coordination, sarbecoviruses downregulate NKG2D ligands (eg, via ORF6 and Nsp1), blunting NK degranulation; restoring those signals rescues NK responses, consistent with display-focused tactics that dampen early coordinated CTL and NK responses [63]. For curated evidence that herpesvirus imprinting can be protective or cross-protective, see Supplementary Material S2.3.

SARS-CoV-2 provides a further test of the framework: This virus reveals how keystone-imprinted memory may be reactivated, redirected, or mislocalized, affecting disease severity and vaccine responsiveness. Further studies have mapped between 30 and 40 immunodominant T-cell epitopes per individual, showing that SARS-CoV-2 immunodominance is dictated by affinity for specific HLA molecules rather than sequence homology alone [64]. Unlike HIV and HCV, which evade immune recognition primarily through antigenic drift, SARS-CoV-2 employs recombination-based strategies that generate novel sub-genomic RNA transcripts while maintaining partial immune recognition [65]. Deep-time analyses emphasize that conserved RNA structures and regulatory motifs can constrain where recombination occurs; peptides encoded in such multilayer-constrained regions may be more robust vaccine targets than those constrained only at the amino acid level [17, 66].

Cross-reactive CD8+ T cells specific for CMV epitopes can also recognize SARS-CoV-2 spike peptides via shared HLA restriction despite low sequence similarity [67], and cross-reactivity between SARS-CoV-2 and seasonal coronaviruses has been documented [68]. In parallel, re-activation of latent herpesviruses (particularly EBV and CMV) was observed in a substantial proportion of hospitalized COVID-19 patients [7], supporting the concept that heterologous immune perturbation can transiently breach viral latency control. In addition, SARS-CoV-2 re-shapes immunity not by altering epitopes but by avoiding compartments where keystone-trained surveillance resides: it suppresses type I and III IFN signaling, evades mucosal tissue-resident memory T cell re-stimulation, and shapes recall toward systemic compartments [69]. Current intramuscular vaccines generate strong systemic memory but generally weaker and less consistent mucosal resident memory than infection, breakthrough infection, or mucosal boosting [69–72]. The practical implication is to prioritize conserved, tissue-relevant targets and delivery that builds tissue-local programs, rather than designs that maximize systemic magnitude while leaving mucosal compartments under-protected. Extended evidence for SARS-CoV-2 immunology, including cross-reactive priming, herpesvirus reactivation, and compartmental evasion, is provided in Supplementary Material S4.

## HETEROLOGOUS IMMUNE PERTURBATION

Latent DNA viruses require continuous immune surveillance to suppress reactivation [5, 73]. Animal models demonstrate that heterologous infection (eg, helminth co-infection) can reactivate latent gamma-herpesviruses via cytokine competition: Reese et al showed that helminth infection reactivates murine gammaherpesvirus (MHV)-68 via interleukin (IL)-4-induced STAT6 signaling, which counteracts IFN-γ-mediated suppression of the viral lytic switch, establishing a “two-signal” model for reactivation [73]. In humans, *Plasmodium falciparum* infection during pregnancy is associated with EBV reactivation [74]. CMV-specific CD8+ T cells expand during unrelated viral challenge (eg, vaccinia infection) through both cross-reactive TCR recognition and TCR-independent cytokine signals such as IL-12 and IL-18 [75]. Barton et al showed that latently infected mice display sustained macrophage activation and protection from unrelated bacterial infections, suggesting that latency may elevate basal immune readiness [5]. Whether receptor-specific or cytokine-driven, this mobilization reflects the host’s ability to harness keystone-imprinted memory in defense against new threats. This functional readiness is consistent with the proposed adaptive benefit of keystone-driven immune imprinting. Extended evidence is provided in Supplementary Material S5.

## TUMOR IMMUNOEDITING THROUGH THE KEYSTONE LENS

We hypothesize that TCRs primed by persistent herpesvirus infections, such as EBV and CMV, may cross-recognize tumor-associated antigens through molecular mimicry. Herpesvirus-specific T cells are known to cross-recognize allogeneic class I HLA molecules [76], and TCR cross-reactivity is a central consideration in cancer immunotherapy [77]. TCRs with suitable peptide specificity can be engineered for use in adoptive cell therapies [78]. Tumors also co-opt the HLA-E/NKG2A axis that gates both CD8 and NK effector programs; single-cell and clinical analyses in bladder cancer show that NKG2A blockade restores cytotoxicity when tumors retain HLA-E and DNAM-1 ligands, underscoring how display-level wiring can be therapeutically reversed [2, 14].

If such mimicry-based surveillance occurs, it could also create vulnerabilities. Tumors that alter class I expression, upregulate inhibitory ligands, or reinforce checkpoint pathways can reduce the effectiveness of cross-reactive T-cell responses, potentially recruiting T-cell infiltration without productive killing [79, 80]. Whether specific tumor neoantigens systematically engage herpesvirus-primed TCRs in non-productive configurations remains to be established; the possibility is consistent with observed patterns of T-cell infiltration without clinical benefit, but alternative explanations including checkpoint-mediated suppression and antigen loss are well documented. The practical implication parallels the RNA virus design principles: prioritize tumor targets that are presented where control is needed, account for antigen display context including the HLA-E/NKG2A axis and combine with strategies that restore presentation balance, so CTL gains translate into coordinated killing. Extended evidence on herpesvirus cross-protection is provided in Supplementary Material S2.3. The 3-stage selection model (thymic selection followed by postnatal keystone focusing) and its implications for modified-self vulnerability are detailed in Supplementary Material S7.2.

For the keystone lens to add explanatory value to tumor immunology beyond established checkpoint and antigen-loss models, 2 conditions would need to hold: (i) keystone (herpesvirus)-primed TCR repertoires contribute measurably to anti-tumor immunity through cross-reactivity, in a way that predicts clinical outcome; and (ii) the pattern of T-cell infiltration without productive killing is attributable specifically to keystone-primed TCRs in non-productive configurations, distinguishable from checkpoint-mediated suppression and antigen loss. We regard both as open, testable questions. Until they are addressed, checkpoint biology and antigen-loss models remain sufficient to explain the tumor observations discussed here, and we present the keystone interpretation as a hypothesis that generates additional, falsifiable predictions (Supplementary Material S6).

## CONCLUSION AND DESIGN RULES

If persistent DNA viruses shape immune priorities within tissue niches as proposed, then rapidly evolving RNA viruses and tumors provide a stringent test: they may divert responses toward mutable or nonproductive targets and rewire antigen display to preserve inhibitory NK signaling while reducing cytotoxic visibility [2].

Three practical design principles follow: (i) target epitopes where substitutions carry measurable fitness costs, (ii) avoid immunogens dominated by mutable targets that inflate magnitude without control, and (iii) normalize antigen display so gains are visible to both CTL and NK arms. Because immunodominance is competitive and finite, these rules address an allocation problem: Boosting magnitude matters less than preventing attention capture by non-productive targets [17].

By display normalization, we mean interventions that restore CTL-relevant pHLA on target cells, making cytotoxic gains visible to both CTL and NK arms. Several candidate levers exist, predominantly in oncology: (i) relief of HLA-E/NKG2A inhibition with NKG2A-blocking antibodies, which can restore cytotoxicity when tumors retain HLA-E [14]; (ii) stabilization of activating NK ligands, for example antibodies that prevent MICA and MICB shedding and thereby sustain NKG2D engagement [81]; and (iii) upregulation of class I and antigen-processing machinery with interferons or epigenetic agents such as histone-deacetylase or DNA-methyltransferase inhibitors [82, 83]. For rapidly evolving RNA viruses such as HIV, by contrast, no established intervention reverses Nef-and Vpu-mediated display remodeling: Here, display normalization is at present a conceptual design goal rather than a deployable strategy, and identifying tractable levers is itself a research priority.

The framework is useful only if integrating epitope constraint, antigen display context, and CTL/NK coordination improves target selection beyond what standard immunodominance reasoning already provides and yields falsifiable predictions.

The most informative experiments directly test the framework’s distinctive claim: that some HLA-associated adapted epitopes preserve detectable T-cell recognition while shifting responding CD8 populations toward less effective states, possibly overlapping receptor space shaped by persistent keystone infections.

**Figure 6.**
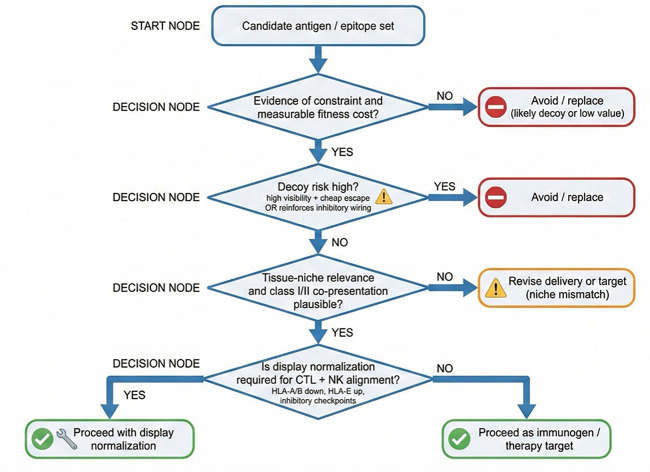
**Practical decision tree for keystone-aware vaccine and immunotherapy design.** Candidate targets are filtered based on evidence of constraint and measurable fitness cost, decoy risk (high-visibility, easily mutable, or reinforcing inhibitory NK signaling), tissue-niche relevance and class I/II co-presentation, and display context (whether normalization is required). Outputs: proceed, proceed with display normalization, or avoid/replace.

Six falsifiable designs follow: (i) paired single-cell profiling of CD8 responses to wild-type vs adapted pHLA, with paired TCR sequencing and direct cytotoxicity readouts, followed by TCR re-expression to map cross-reactivity with candidate keystone-derived epitopes; (ii) immunogen comparisons scored on breadth, immunodominance structure, and functional quality (cytotoxicity and polyfunctionality) rather than IFN-γ magnitude alone; (iii) population-level 4-category analysis of viral load (HLA present or absent crossed with wild-type or adapted residue), testing for sites where the adapted residue tracks with higher viral load specifically in HLA-matched hosts who retain recognition; (iv) natural-processing and display-context validation, with experimental manipulation of Nef/Vpu class I modulation and the HLA-E/NKG2A axis; (v) comparative analysis of class I modulation across unrelated pathogens and tumors; and (vi) perturbation studies of latency control using the Keystone Reactivation Index as a longitudinal metric. Assay-level protocols and citations for each are given in Supplementary Material S6. A practical decision tree for this workflow is provided in Figure 6.

## SUPPLEMENTARY DATA

Supplementary materials are available at the *Pathogens and Immunity* website. Supplementary data may be provided by the authors to benefit the reader. Supplementary data are not copyedited and are the sole responsibility of the authors. Questions or comments related to supplementary materials should be addressed to the corresponding author.
