# Supplementary material for: Keystone Epitope Theory: An Ecological Perspective on RNA Viruses, Tumor Immunoediting, and Vaccine Design
Source: Pathog Immun. 2026 Aug 7;11(2):39–65. doi: 10.20411/pai.v11i2.1011 (PMC13471027; doi:10.20411/pai.v11i2.1011)
Supplement: Supplementary file 1 [file pai-11-039-s01.pdf]

PATHOGENS AND IMMUNITY
IN ASSOCIATION WITH CASE WESTERN RESERVE UNIVERSITY AND THE RICHARD J. FASENMYER FOUNDATION

SUPPLEMENTARY MATERIALS

S1. Table 1. Expanded Glossary and Conceptual Clarifications

Key terms used in this review. Model constructs are explicitly hypotheses; phenomenon, mechanism, and method terms are used as defined in the cited literature.

Term	Definition
**Keystone Epitope Theory (KET)**	Model/framework: a subset of persistent, human-adapted infections disproportionately shapes immune priority-setting by repeatedly restimulating specific peptide–HLA targets in tissue niches. A falsifiable hypothesis, not a settled mechanism.
**Keystone organism**	Persistent, human-adapted infection that (i) establishes latency or chronic low-level replication, (ii) repeatedly restimulates memory in defined tissue niches, and (iii) presents conserved, function-linked epitopes; the claim is an outsized effect on immune architecture, not a binary class.
**Keystone epitope**	A peptide–HLA target that is repeatedly presented (persistence/reactivation), constrained (adaptation limited or costly), and linked to durable, high-quality T-cell and sometimes coordinated humoral responses.
**Keystone imprinting**	Durable biasing of immune priorities established by early, repeated keystone exposure; broader than original antigenic sin, which is variant-strain skewing within a viral family.
**Immunodominance**	Umbrella term, used here only with a modifier because distinct phenomena are often conflated: dominance may refer to display, response magnitude, temporal dynamics, population frequency, anatomical compartment, or clinical control.
**Presentation-dominance**	A peptide abundant or stable on a given HLA molecule (HLA peptidomics); does not guarantee a dominant T-cell response.

Mallal S, Asiaee A. Keystone Epitope Theory: An Ecological Perspective on RNA Viruses, Tumor Immunoediting, and Vaccine Design. *Pathogens and Immunity*. 2026;11(2):39-65. doi: 10.20411/pai.v11i2.1011

Term	Definition
**Episode-specific**	Rank order of epitopes by response magnitude or function at one timepoint in one host.
**Longitudinal**	Within-host hierarchy that changes over time (for example, adaptation-driven collapse with subdominant fill-in).
**Compartmental**	Dominance defined within an anatomical compartment (for example, tissue-resident memory-rich tissue versus blood).
**Control-dominance**	Disproportionate contribution to pathogen control or clinical outcome, independent of magnitude or prevalence.
**Immunoprevalence**	Proportion of a defined cohort mounting a detectable response (assay and threshold stated); used here for the population-level "how many respond" component.
**Immunodominance hierarchy**	An ordered list of targets plus the metric and level used; a data summary that implies no mechanism.
**Constrained epitope**	An epitope where substitutions incur measurable fitness costs (replication, transmission, structural integrity), limiting or penalizing adaptation.
**Decoy epitope**	An epitope eliciting strong or sustained responses that contribute little to control, often because it is mutationally permissive or poorly positioned in time or tissue.
**Antigen display control**	Viral or tumor strategies that alter peptide–HLA output and co-ligands so CTL visibility falls while inhibitory NK signaling is preserved or enhanced; more than antigenic variation alone.
**Shared CTL–NK vulnerability**	CTLs and NK cells read overlapping HLA class I with different receptor logic; successful adaptation requires selective remodeling that lowers CTL-relevant pHLA without triggering NK rescue (via HLA-C/KIR, HLA-E/NKG2A, licensing, or checkpoints).

Term	Definition
**CTL visibility; inhibitory NK signaling**	CTL visibility: ease of CTL detection via relevant pHLA. Inhibitory NK signaling: net inhibitory NK input (HLA-C, HLA-E/NKG2A, peptide-dependent KIR, licensing, checkpoint) that can block NK killing despite cellular stress.
**Peptide–HLA (pHLA)**	The cell-surface complex of a peptide bound to an HLA molecule; the recognition unit for CD8 T cells and an input to some NK receptors.
**TCR**	Somatically rearranged T-cell receptor that recognizes specific peptide–HLA complexes.
**KIR**	NK immunoglobulin-like receptors that bind HLA class I; some interactions are peptide-influenced, tuning inhibition or activation.
**NKG2A / HLA-E**	Inhibitory NK checkpoint axis: NKG2A (with CD94) recognizes HLA-E presenting leader-like peptides, contributing to NK inhibition and calibration.
**TRM**	Tissue-resident memory T cells that persist long-term in tissues rather than recirculating.
**Tumor immunoediting**	Tumor evolution under immune selection, often favoring antigen loss, altered processing or presentation, altered HLA expression, and checkpoint reinforcement.
**Immune checkpoint**	Inhibitory pathways that restrain immune activation, including reinforcement that preserves inhibitory signaling under cellular stress.
**Modified self**	A self-derived peptide rendered newly immunogenic when a drug or post-translational modification alters processing or the peptide–HLA repertoire, potentially recruiting pre-existing memory.

Term	Definition
**Niche-calibrating epitope**	A recurrent peptide–HLA surface displayed in a defined niche by a persistent antigen source that tunes local thresholds of cytotoxicity, regulation, and recall; keystone epitopes are the apex subset [1, 2].
**Equilibrium phenotype**	A regulated differentiation state often labeled "exhaustion," with checkpoint expression and restrained effector output that supports long-term containment under persistent antigen [1].
**Four-Condition Gate**	Model: severe immunopathology requires conjunction of permissive HLA binding, a provoking exposure or modification, pre-existing cross-reactive structural memory, and niche-matched antigen display [1, 3].
**Keystone Reactivation Index (KRI)**	Candidate longitudinal metric: the fraction of microbial detections in a compartment and time window attributable to keystone organisms, with detection type and assay threshold pre-specified (for example, CMV, EBV, HSV in blood, bronchoalveolar fluid, or gut mucosa); not yet prospectively validated [4].
**Epitope / paratope**	Used in an operational, receptor-dependent sense: the antigen features contributing to binding and recognition by a given receptor in context, not a fixed, context-free surface patch [5, 6].

Usage notes: "Immunodominant" means dominant by a stated metric, not most protective. "Integrate into immune hierarchies" refers to durable positioning within immune memory, not viral DNA integration. Agency verbs ("exploit," "optimize," "designed") denote selection effects, not intent. "Linkage" (chromosomal distance) is distinct from linkage disequilibrium (LD), which we name explicitly. Abbreviations: CTL, cytotoxic T lymphocyte; CMV, cytomegalovirus; EBV, Epstein-Barr Virus; HSV, Herpes Simplex Virus; KIR, Killer cell immunoglobulin-like receptor; NK, natural killer; TCR, T-cell receptor

S2. Supplementary Boxes

S2.1. Box 1. Primer to Keystone Epitope Theory

(A summary of the KET framework as developed in the main text)

Keystone Epitope Theory adds a third, postnatal focusing step (Supplementary Material S7.2) to immune education. This same postnatal window also solves barrier onboarding (the postnatal process by which mucosal and skin surfaces establish tolerance to environmental antigens): rapidly rising antigen exposure at mucosal and skin surfaces drives active peripheral tolerance (pTregs, tolerogenic antigen-presenting cell conditioning, IgA), while keystone niches remain high-gain channels that are repeatedly re-engaged across life (Figure 3 in the main text). After thymic positive/negative selection, a small set of persistent, human-adapted organisms concentrates immune memory on constrained, function-linked epitopes in the tissues where control must occur. This imprinting is protective because it hardwires priorities on targets the host cannot afford to miss, yet it also defines where the modified self can recruit entrenched memory (Supplementary Material S7.2).

Keystone equilibrium is a distinct "middle regime." Keystone control is typically neither peripheral tolerance nor sterilizing clearance. It is a stable, tissue-compartmentalized equilibrium that preserves high-gain recall to constrained epitopes in the econiche where control matters, while avoiding chronic tissue damage. Immune resources are finite, and responses directed toward one set of targets necessarily reduce the capacity to respond to others. In this framing, equilibrium is an allocation under finite tissue and metabolic constraints, not a failed sterilization attempt [1]. Its stability depends on niche-specific inhibitory set points, which is why immune checkpoint blockade can precipitate tissue-specific injury without introducing new antigens [2].

Stage 3 addresses 2 problems simultaneously: keystone focusing and barrier onboarding. In parallel with keystone focusing, early postnatal antigen exposure at the gut, skin, and airway surfaces establishes active tolerance programs (eg, pTreg induction, secretory IgA-based exclusion, tissue-conditioned myeloid restraint) that prevent abundant, low-salience exposures from becoming chronic inflammatory drivers. When onboarding fails, benign antigens are repeatedly encountered in danger contexts (dysbiosis, barrier breach, viral inflammation). They can be misclassified as pseudo-keystones, thereby distorting subsequent immunodominance and

risk of pathology. Beyond keystone epitopes, persistent niche-restricted residents can also dominate local display and tune thresholds; we refer to their recurrent peptide–HLA targets as niche-calibrating epitopes [1, 2].

These priorities are read jointly by cytotoxic T cells and NK cells on overlapping class I ligand landscapes. Simple loss of class I can release NK killing, but pathogens such as HIV can more effectively blunt both arms by selective remodeling: reducing HLA-A/B to weaken cytotoxic T lymphocytes (CTLs) while variably tuning HLA-C, preserving HLA-E/NKG2A signaling, exploiting peptide-dependent killer cell immunoglobulin-like receptor (KIR) effects, and reinforcing checkpoint pathways [7]. The net result is a host- and strain-dependent shift in the balance of CTL and NK pressure rather than automatic uncoupling.

Persistent DNA viruses (notably HHVs) scaffold durable memory (ie, provide a structural framework for organizing and maintaining long-lived immune memory): they seed tissue-resident compartments, re-stimulate at low levels to maintain recall, and maintain Class I/II epitopes in appropriate anatomical niches at appropriate times. This temporal–spatial layering explains why some targets remain longitudinally immunodominant throughout life and why niche-matched display later in life matters for pathology.

The same architecture (the structural organization of immune memory and priorities) creates a window of vulnerability. When a drug- or post-translational-modified self-peptide approximates a keystone epitope and is displayed in the same tissue by the same risk HLA, pre-existing memory may engage under a thresholded control regime, and can drive pathology if mimicking ligand burden exceeds local inhibitory set-points [1, 2]. The same gate-and-threshold logic provides a compact explanation for why many HLA risk alleles have low positive predictive value, since permissive binding is necessary but rarely sufficient without niche-matched display and memory-backed amplification. That is the mechanistic bridge from keystone protection to T-cell-mediated hypersensitivity; the clinical evidence is detailed in a companion review [8].

Selection pressures have favored HLA– T-cell receptor (TCR) solutions that see conserved, functionally costly viral sites, while still permitting cross-reactivity that anticipates related threats. The allelic logic (eg, HLA-B57/B58) and the structural/conformational basis for public and private TCR cross-recognition are outlined in a companion review [8].

Because keystone organisms train memory in place, tissue compartmentalization matters. Tissue-resident memory (TRM) at the dermal–epidermal junction, in lymphoid tissue, or along the vasculature can be recruited directly by parenchymal display, a key insight for patterned injury in skin, lymphoid organs, and entheses.

Timing and sequence of early-life exposures may matter. Variation in when and in what order infants encounter persistent keystone organisms could, in principle, alter subsequent immune calibration. This is a hypothesis about exposure timing and sequence, not an argument against any intervention: vaccination, antibiotics, and sanitation remain overwhelmingly beneficial. Where early exposure is delayed or reordered, KET predicts that subsequent immune hierarchies may be calibrated differently, potentially altering how later challenges are handled [9]. Discordant co-evolution (host immunogenetics meeting non-co-adapted pathogen strains) may add another axis of variation.

Keystone exposure also shapes heterologous immunity: persistent herpesviruses can enhance cross-protective responses and modulate regulatory and innate immune programs (including NK cells). These effects are protective but context- and genotype-dependent, reinforcing the need to read immunity as a coordinated system.

The present paper analyzes RNA viruses and tumors as stringent tests of the system: decoy vs constrained epitopes, antigen display control (eg, HLA-E/NKG2A), and design rules for vaccines and immunotherapies that work with, rather than against, entrenched hierarchies. A companion review [8] develops the clinical translation to drug hypersensitivity, autoimmunity, and transplantation.

S2.2. Box 2. Examples of Keystone-Like Vaccine Imprinting

The live attenuated varicella-zoster virus (VZV) and yellow fever virus (YFV) vaccines discussed here can exhibit keystone-like properties: their replication kinetics, tissue tropism, and synchronized class I and II epitope presentation during early infection induce durable CD8+ and CD4+ T-cell memory [10, 11], delivering antigen in the anatomical compartments and temporal windows characteristic of natural infection. The two vaccines differ in persistence biology, but both can produce effective immune imprinting with substantially less morbidity than natural infection and therefore provide useful, although not identical, examples of keystone-like immune calibration.

S2.3. Box 3. Evidence of Human Herpesvirus Protection or Cross-Protection

Multiple lines of evidence indicate that herpesvirus exposures can precondition immune hierarchies. Within the family, an HSV-2 glycoprotein vaccine protects mice against genital HSV-1 challenge [12]. In humans, HSV-1 acquisition varies substantially by age and region, contributing to marked geographic variation in the estimated burden of genital HSV-1 infection [13]. In murine models, maternal immunization with HSV antigens reduces neonatal mortality and neurological morbidity through passive antibody transfer and neonatal innate priming [14], and conserved-glycoprotein (gD) vaccines confer cross-protection against both HSV-1 and HSV-2 in guinea pigs [15]. Imprinting also extends to unrelated pathogens: latent murine γ-herpesvirus 68 or murine cytomegalovirus increases resistance to *Listeria monocytogenes* and *Yersinia pestis* through sustained interferon (IFN)-γ and macrophage activation [16], lifelong cytomegalovirus (CMV) broadens the TCR repertoire mobilized against third-party *Listeria* in aged mice [17], and in humans CMV- and EBV-specific CD8 T cells are recruited into the activated pool during acute hepatitis B [18, 19]. Host genetics also contribute: activating KIR gene content has been associated with reduced CMV infection/reactivation after transplantation, and KIR3DS1 in combination with HLA-B Bw4-80I has been associated with delayed HIV disease progression [2, 20, 63]. Together, these findings support cross-protection through cross-reactive recognition, cytokine-driven bystander activation, or altered antigen presentation, although the mechanisms remain incompletely defined.

S3 EXTENDED HIV EVIDENCE

S3.1. HIV as an Engine of Immune Adaptation

HIV's rapid within-host evolution makes it a powerful driver of immune selection. Mutations within immunodominant epitopes often persist in the population despite immune pressure because they impose little fitness cost [21], and codon-usage analysis suggests HIV is pre-adapted to respond rapidly to HLA-specific pressure and to generate decoy epitopes [22]. Rather than evading recognition entirely, some substitutions may redirect CD8+ responses toward ineffective targets, though this requires epitope-level functional validation [21, 23-26]. A deep-time extension of KET treats synonymous and RNA-structural constraints as an active substrate for adaptation, so epitope constraint should be evaluated at both peptide and RNA layers [1, 27].

S3.2. Decoy Epitopes and Immune Misdirection

A direct consequence is preferential inflation of Nef-directed responses at the expense of more protective Gag-specific responses [24]. At the population level, HIV substitutions within CTL epitopes associate consistently with specific HLA alleles [28]; such associations do not by themselves distinguish recognition-reducing adaptation, in which selection removes recognition and which tends to occur preferentially at HLA-binding residues of CD8 epitopes [29], from adaptation that preserves detectable recognition while reducing antiviral function, the latter remaining a narrow, testable possibility. Chronic-stimulation-driven functional impairment is an alternative explanation, and discrimination requires paired wild-type versus adapted comparisons within individuals using direct cytotoxicity and single-cell profiling [21, 26, 30].

S3.3. Population-Level Viral Adaptation and Pre-adaptation

HIV evolves differently across populations, with distinct HLA-adapted polymorphisms in genetically distinct host groups [31]: where a dominant HLA allele consistently targets an epitope, recognition-reducing substitutions are favored, whereas greater HLA diversity favors decoy substitutions that divert responses from constrained regions at low fitness cost. Across transmissions, host hierarchies originally calibrated by keystone organisms shape the selective landscape; transmission of HLA-adapted variants weakens subsequent responses and is associated with higher viral loads and faster CD4+ decline [32], while the protective value of targeted alleles erodes over time [33]. Epistatic host factors such as ERAP2 further modulate the presented peptide repertoire and adaptation [34].

S3.4. HLA-Associated Substitution and Reversion Dynamics and Evolutionary Lock-In

HIV adaptation involves both substitution and reversion: HLA-B57-associated substitutions revert rapidly in non-B57 hosts, whereas other HLA-associated mutations remain stable [25]. Persistence in the absence of continued immune pressure can reflect compensatory mutations that stabilize a locked-in state across a fitness valley, allowing such polymorphisms to keep misdirecting responses.

S3.5. Implications for HIV Vaccine and Drug Resistance

Not all HLA-associated substitutions impose a fitness cost, and some are selected to misdirect responses; these polymorphisms can also affect prevention, for example, HLA-B18-driven substitutions at reverse transcriptase codon 138 linked to natural rilpivirine resistance [35], with immune and drug-resistance selection pressures intersecting to shape diversity [36]. HLA-II-associated adaptation weakens CD4+ help in vaccine recipients, plausibly reflecting a mismatch between vaccine-induced immunodominance and keystone-based hierarchies that, via linked recognition, favor high-affinity T- and B-cell clones; without persistent antigenic reinforcement, responses may skew toward immunogenic but poorly protective epitopes with inadequate T follicular helper coordination [37]; these constraints underscore the broader design challenges facing antibody-based HIV-1 vaccines [38]. Early CTL responses drive adaptation even before seroconversion [39], and accumulation of substitutions at formerly protective alleles (HLA-B57, HLA-B27) has eroded their control [40]. Integrase illustrates the constraint principle: integrase inhibitors succeed by targeting catalytic residues that cannot mutate without loss of function, whereas TCR recognition is more vulnerable because the virus can mutate peripheral residues outside the functional pocket; consistent with this, HLA-driven substitutions were not observed at primary integrase resistance sites, only at non-primary sites [41]. More broadly, DNA viruses act as persistent calibrators while RNA viruses act as disruptors using rapid variation and decoys [42]; HLA-B targeting efficiency for conserved regions tracks with outcome, with efficient Gag targeting associated with lower HIV viral load [43] and dengue HLA targeting efficiency correlating with severity [42]. Because RNA viruses rarely provide the persistent antigenic scaffolding needed to select and maintain high-affinity clones against conserved targets, durable high-fidelity immunity is harder to generate by infection or vaccination.

 Mallal S, Asiaee A. Keystone Epitope Theory: An Ecological Perspective on RNA Viruses, Tumor Immunoediting, and Vaccine Design. *Pathogens and Immunity*. 2026;11(2):39-65. doi: 10.20411/pai.v11i2.1011

S3.6. Conservation vs Constraint in Immunogen Design

Sequence conservation is descriptive and is not equivalent to mechanistic constraint: it can arise from protein-level fitness costs, RNA-structural requirements, historical HLA-driven adaptation, or context-dependent processing and display. Rolland et al found no strong relationship between consensus-residue frequency and replicative capacity and argued for prioritizing sites that remain conserved *despite* immune pressure [44]; the HIVconsvX design reflects this logic, excluding conserved regions linked to unfavorable outcomes and drawing four of six regions from Pol despite its low expression [45]. This material is developed in the main text ("Decoy vs constrained epitopes").

S4. EXTENDED SARS-COV-2 EVIDENCE

S4.1. Pre-Existing Cross-Reactive Memory and Keystone Priming

Unlike persistent DNA viruses or highly mutable RNA viruses, SARS-CoV-2 engages immune memory pre-structured by earlier coronavirus exposures: 20–50% of unexposed individuals have detectable T-cell reactivity against SARS-CoV-2 [46], largely from common-cold coronaviruses sharing homologous epitopes. Immunodominance is set by epitope-HLA affinity rather than sequence homology alone, with 30–40 immunodominant epitopes mapped per individual [47], and a survey of common-cold coronaviruses identified 165 CD4+ epitopes, many also recognized in SARS-CoV-2, with 89% of cross-reactivity predicted by greater-than-67% sequence conservation [48]. These patterns support a central tenet of Keystone Epitope Theory: early exposures shape durable, hierarchical memory that influences responses to later-emerging pathogens, even across genera.

S4.2. Rewiring Immunodominance: Beyond Sequence Homology

Epitope hierarchy is shaped by HLA-binding affinity, recombination, and immunogen structure rather than sequence similarity alone. Unlike HIV and HCV, SARS-CoV-2 uses recombination-based strategies that generate novel sub-genomic transcripts while preserving partial recognition [49]; conserved RNA structures can constrain where recombination occurs, so peptides in multi-layer-constrained regions may be more robust vaccine targets than those constrained only at the amino-acid level [1, 27]. As for other RNA viruses, effective vaccines should prioritize stable, functionally constrained regions over immunodominant but mutable targets [42], which may help explain the historical difficulty of developing protective RNA-virus vaccines.

 Mallal S, Asiaee A. Keystone Epitope Theory: An Ecological Perspective on RNA Viruses, Tumor Immunoediting, and Vaccine Design. *Pathogens and Immunity*. 2026;11(2):39-65. doi: 10.20411/pai.v11i2.1011

S4.3 Latent Herpesvirus Reactivation and Immune Disruption

Acute SARS-CoV-2 infection can destabilize pre-existing memory. In the IMPACC cohort, reactivation of latent EBV and CMV occurred in a substantial proportion of hospitalized patients (EBV early; CMV and HSV-1 later, correlating with interleukin (IL)-6 and CXCL10), consistent with transient epitope-triggered activation of latent DNA viruses under immune perturbation [50]. SARS-CoV-2-reactive T cells occur in unexposed individuals [46], and SARS-CoV-2 mRNA vaccination induces CD4+ T-cell responses that cross-recognize HCoV-NL63 [51]. Separately, a public, HLA-B*35:01-restricted CD8 TCR recognizes both a CMV pp65 epitope (IPSINVHHY) and a SARS-CoV-2 spike peptide (FVSNGTHWF) despite low sequence similarity and can reduce replication in vitro, though it is weakly activated in acute disease [52]. Notably, pp65, although strongly immunodominant, is dispensable for CMV persistence [53], underscoring that immunodominance need not coincide with the antigens most essential to the organism. Further CD8+ T-cell cross-reactivity is documented for an HLA-B*07:02-restricted, immunodominant SARS-CoV-2 nucleocapsid epitope with homologous epitopes from the seasonal betacoronaviruses OC43 and HKU1, but not the alphacoronaviruses 229E or NL63 [54]. A companion study showed that B7/N105-specific CD8+ T cells have high naive precursor frequency, broad TCRαβ diversity, and promiscuous TCR pairing, with pre-pandemic B7/N105-specific cells predominantly displaying a naive phenotype rather than clear recall memory [55]. Separately, uninfected donors can harbor SARS-CoV-2-reactive T cells targeting N, NSP7, and NSP13; NSP7-specific responses recognize fragments conserved among animal betacoronaviruses but with low homology to common-cold human coronaviruses [56]. Direct T-cell cross-reactivity with EBV, HHV-6, or HSV is not established by these references. Keystone Epitope Theory predicts that high-frequency naive or cross-reactive pools recruited by early immunodominant SARS-CoV-2 epitopes could transiently compete with more effective subdominant responses.

S4.4 Compartmental Misdirection and IFN Evasion

SARS-CoV-2 suppresses type I and III interferon signaling via multiple nonstructural proteins, delaying viral recognition and T-cell priming [57]. Respiratory TRM T cells, which would otherwise trigger rapid interferon release on detecting infected cells, appear to be evaded;

 Mallal S, Asiaee A. Keystone Epitope Theory: An Ecological Perspective on RNA Viruses, Tumor Immunoediting, and Vaccine Design. *Pathogens and Immunity*. 2026;11(2):39-65. doi: 10.20411/pai.v11i2.1011

plausibly SARS-CoV-2 and endemic coronaviruses avoid re-stimulating mucosal TRM and shape recall toward systemic compartments and decoy antigens, consistent with current vaccines generating strong circulating memory but few TRM [57]. This compartmental misdirection may transiently re-engage keystone-shaped immunity while evading the interferon alarms that would abort early replication, destabilizing imprinted memory and favoring latent reactivation. Keystone Epitope Theory is thereby refined: memory success depends not only on epitope specificity but also on anatomical accessibility, so even well-imprinted memory can fail if mislocalized.

S5. Extended Heterologous Immune Perturbation Evidence

Animal models support a dynamic view of latency in which latent DNA viruses require continuous immune surveillance. Barton et al. showed that CD8+ T cells and IFN-γ control murine gammaherpesvirus (MHV)-68 latency, and that latently infected mice display sustained macrophage activation and protection from unrelated bacterial infection, suggesting latency may elevate basal immune readiness [16]. Reese et al found that helminth infection reactivates MHV-68 via IL-4/STAT6 signaling that counteracts IFN-γ suppression of the lytic switch, a two-signal model [58], and in humans *Plasmodium falciparum* infection during pregnancy was associated with EBV reactivation [59], indicating that heterologous stimulation can transiently breach latency control. Such transient reactivation may be a functional feature rather than a failure, allowing persistent viruses to act as sentinels that recalibrate immunity to new threats. Gordon et al showed that CMV-specific CD8+ T cells expand during unrelated (vaccinia) challenge without cognate antigen [60], consistent with cytokine-driven bystander activation (IL-12, IL-18) as well as, or instead of, direct TCR-mediated cross-reactivity; these models are distinct but not mutually exclusive, and either way reflect the host's use of keystone-imprinted memory in defense of new threats.

S6. Framework Additions and Experimental Test Protocols

The 3 framework additions and the 6 falsifiable experimental designs are now presented in the main text (Conclusion and Design Rules; see also the 3 considerations summarized in "Keystone Epitope Theory in Brief"). This section retains assay-level detail for each design.

1. **S6.1 Paired single-cell profiling and cross-reactivity mapping.** For HLA-matched donors with well-defined adapted variants, isolate CD8 T cells against matched wild-type and adapted pHLA multimers and profile by single-cell RNA-seq with paired TCR sequencing, CITE-seq, and cytotoxicity readouts (CD107a, target killing); then re-express dominant TCRs in a common background (eg, Jurkat 76 or transduced primary CD8) to test cross-recognition of candidate keystone-derived epitopes selected by shared HLA restriction and TCR-facing pHLA geometry [7, 26].

2. **S6.2 Immunogen comparisons.** Compare constrained-region immunogens against constructs that include variable immunodominant targets, scoring breadth, immunodominance structure, natural processing, and cytotoxic quality rather than IFN-gamma magnitude alone; promote to a primary endpoint only after the mechanistic studies in S6.1 and S6.3.

3. **S6.3 Population-level four-category viral-load analysis.** Across groups defined by HLA present or absent crossed with wild-type or adapted residue, test for sites where the adapted residue is associated with higher viral load specifically in HLA-matched hosts who retain recognition, adjusting for cohort, ancestry, sex, clade, calendar time, linkage, and host antigen-processing modifiers, with multiple-testing correction and replication across clades; chronic-stimulation-driven impairment is the principal alternative and requires single-cell discrimination (S6.1) [31, 32, 61].

4. **S6.4 Natural processing and display-context validation.** Confirm candidate epitopes are naturally processed and displayed (immunopeptidomics, peptide–HLA stabilization, infected-cell killing) and manipulate Nef/Vpu class I modulation and the HLA-E/NKG2A axis to test whether target value depends on coordinated CTL and NK pressure; proof-of-principle exists in oncology [7, 62].

5. **S6.5 Comparative class I modulation across pathogens and tumors.** Using standardized measurements of class I surface expression and NK-receptor engagement, test whether unrelated pathogens and tumors recurrently reduce HLA-A/HLA-B presentation while preserving inhibitory signaling through other class I ligands.

6. **S6.6 Perturbation studies of latency control.** Track the KRI (fraction of microbial detections attributable to keystone organisms in a compartment and time window) across baseline, perturbation, and recovery phases, with peripheral memory-phenotype correlates.

RNA-layer caveat. Synonymous codon usage and local RNA structure may also influence processing efficiency at apparently conserved amino-acid positions; the relevant test is synonymous recoding of candidate epitopes and flanking regions with measurement of viral fitness, peptide generation, and surface pHLA abundance, which does not by itself distinguish decoying from recognition-preserving adaptation.

S7. SUPPLEMENTARY FIGURES

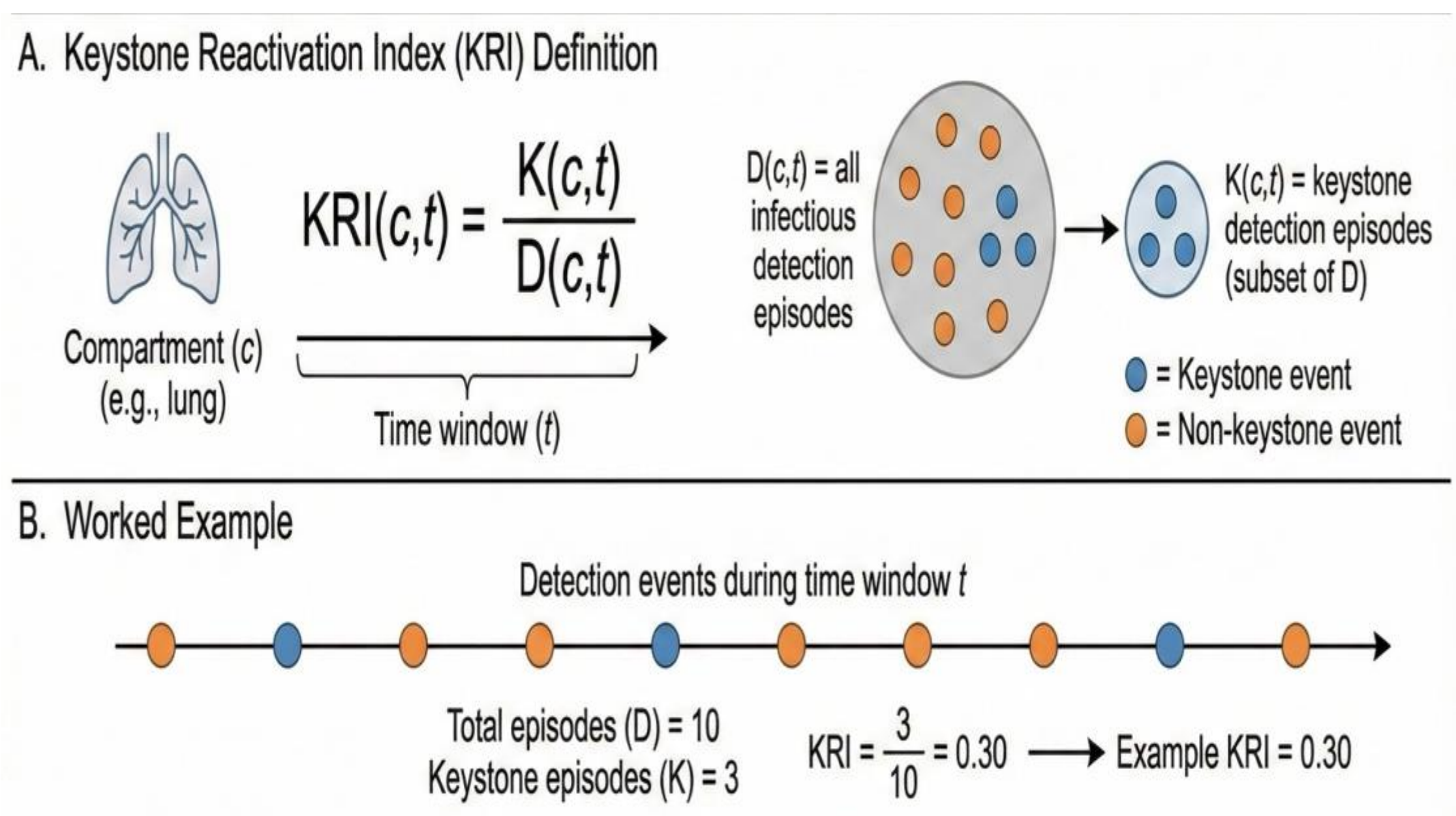

S7.1. Keystone Reactivation Index (KRI): definition and worked example. (A) KRI is defined for a compartment c and time window t as the number of keystone detection episodes divided by all microbial detection episodes, $\text{KRI}(c,t) = K(c,t)/D(c,t)$. (B) Worked example: of ten detection episodes in the window, three are keystone, giving $\text{KRI} = 0.30$. The KRI is a candidate metric only; prospective studies would be required to determine whether longitudinal KRI shifts track immune perturbation in accessible compartments such as blood, bronchoalveolar fluid, or gut mucosa.

 Mallal S, Asiaee A. Keystone Epitope Theory: An Ecological Perspective on RNA Viruses, Tumor Immunoediting, and Vaccine Design. *Pathogens and Immunity*. 2026;11(2):39-65. doi: 10.20411/pai.v11i2.1011

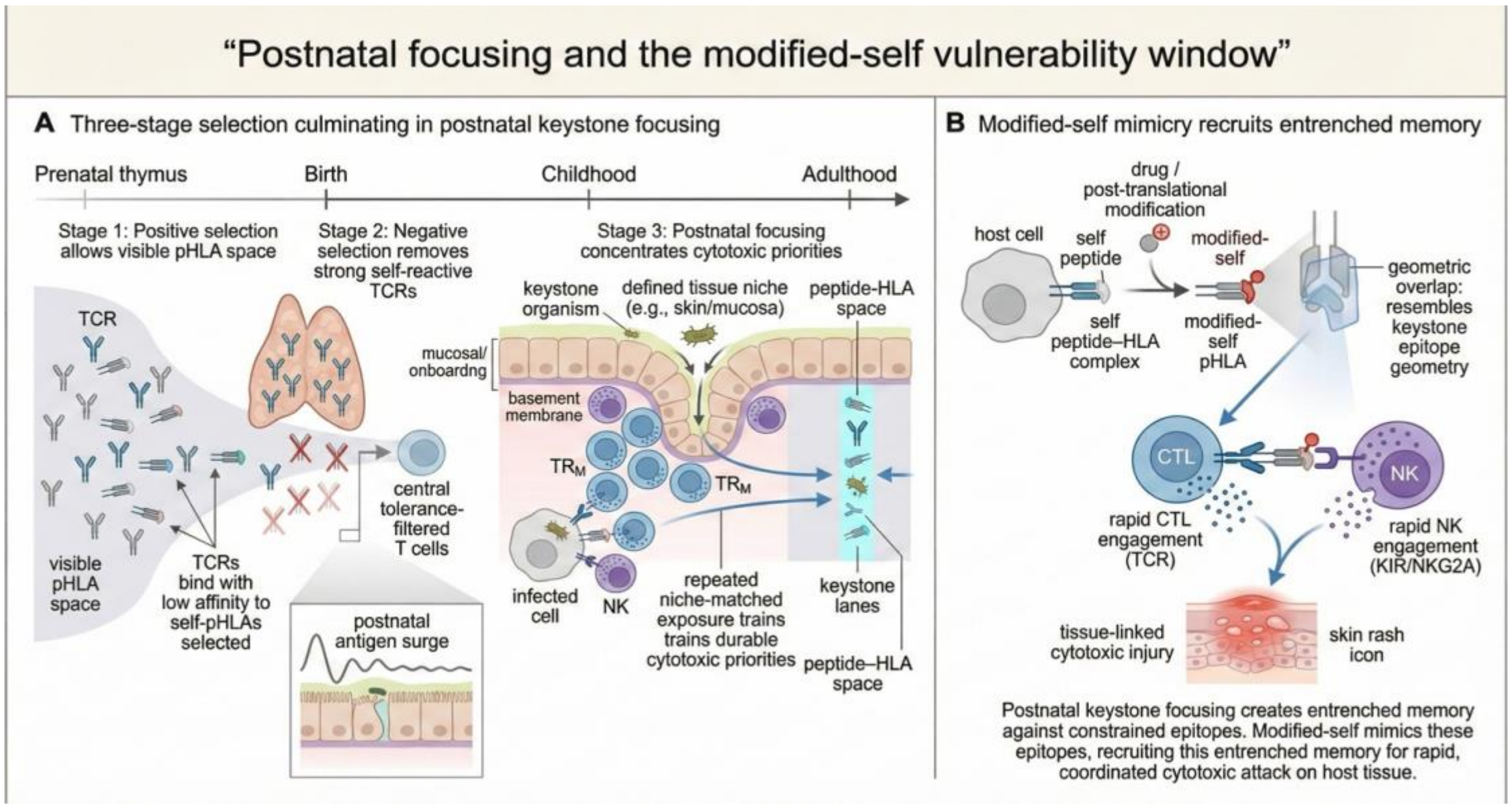

S7.2. Postnatal focusing and the modified-self vulnerability window. (A) Three-stage selection: thymic positive/negative selection defines visible tolerated pHLA space; postnatal, tissue-linked keystone exposure concentrates tissue-resident memory and cytotoxic priorities onto constrained, function-linked epitopes. (B) Modified-self mimicry: drug- or PTM-derived self-peptides that overlap keystone geometry and are displayed in the same tissue niche recruit entrenched memory and trigger rapid CTL/NK injury; the concept is developed in a companion review [8].

SUPPLEMENTARY REFERENCES

1. Mallal S, Phillips E. The Immune Attention Economy: Keystone Epitope Theory and the Strategic Allocation between Tolerance, Equilibrium, and Sterilization. *Zenodo*. 2026. doi: 10.5281/zenodo.18520553.

2. Mallal S. Dynamic Peripheral Tolerance as Niche Control: A Keystone Epitope Theory Synthesis for Natural Autoimmunity and Immune Checkpoint Inhibitor Toxicities. *Zenodo*. 2026. doi: 10.5281/zenodo.18520695.

3. Mallal S, Asiaee A, Mallal N, Phillips E. Keystone Epitope Theory: Implications for the Investigation of Multiple Sclerosis. *Zenodo*. 2026. doi: 10.5281/zenodo.18520803.

4. Asiaee A, Mallal N, Phillips E, Mallal S. Co-Evolved Partners of Immunity: A Trait-Based Map of Human Keystone Organisms. *bioRxiv*. 2025. doi: 10.1101/2025.08.19.671142.

5. Greenspan NS. Epitopes, Paratopes, and Other Topes 30 Years On: Understanding What We Are Talking About. *Hum Immunol*. 2023;84:429-38. doi: 10.1016/j.humimm.2023.06.006.

6. Greenspan NS, Di Cera E. Defining Epitopes: It's Not as Easy as It Seems. *Nat Biotechnol*. 1999;17:936-7. doi: 10.1038/13590.

7. Mallal S, Asiaee A. Keystone Theory: Implications for Effective T and Natural Killer Cell Interoperability. *Zenodo*. 2025. doi: 10.5281/zenodo.17087092.

8. Mallal S, Phillips E. Keystone Epitope Theory: Implications for Hypersensitivity, Autoimmunity and Transplantation. *Zenodo*. 2025. doi: 10.5281/zenodo.17082090.

9. Greenspan NS. Clinical Consequences of Human Evolution Shaped by Cultural Trends. *Evol Med Public Health*. 2013;2013:24-6. doi: 10.1093/emph/eos006.

10. Sei JJ, Cox KS, Dubey SA, Antonello JM, Krah DL, Casimiro DR, Vora KA. Effector and Central Memory Poly-Functional CD4+ and CD8+ T Cells Are Boosted Upon Zostavax® Vaccination. *Frontiers in Immunology*. 2015;6:1-15. doi: 10.3389/fimmu.2015.00553.

11. James EA, LaFond RE, Gates TJ, Mai DT, Malhotra U, Kwok WW. Yellow Fever Vaccination Elicits Broad Functional CD4+ T Cell Responses That Recognize Structural and Nonstructural Proteins. *Journal of virology*. 2013;87:12794-804. doi: 10.1128/jvi.01160-13.

12. Egan KP, Hook LM, Naughton A, Pardi N, Awasthi S, Cohen GH, Weissman D, Friedman HM. An HSV-2 Nucleoside-Modified mRNA Genital Herpes Vaccine Containing Glycoproteins gC, gD, and gE Protects Mice against HSV-1 Genital Lesions. *PLOS Pathogens*. 2020. doi: 10.1371/journal.ppat.1008795.

13. Looker KJ, Magaret AS, May MT, Turner KME, Vickerman P, Gottlieb SL, Newman LM. Global and Regional Estimates of Prevalent and Incident Herpes Simplex Virus Type 1 Infections in 2012. *PloS one*. 2015;10:e0140765. doi: 10.1371/journal.pone.0140765.

14. Patel CD, Backes IM, Taylor SA, Jiang Y, Marchant A, Pesola JM, Coen DM, Knipe DM, Ackerman ME, Leib DA. Maternal Immunization Confers Protection against Neonatal Herpes Simplex Mortality and Behavioral Morbidity. *Sci Transl Med*. 2019. doi: 10.1126/scitranslmed.aau6039.

15. Bourne N, Bravo FJ, Francotte M, Bernstein DI, Myers MG, Slaoui M, Stanberry LR. Herpes Simplex Virus (HSV) Type 2 Glycoprotein D Subunit Vaccines and Protection against Genital HSV-1 or HSV-2 Disease. *J Infect Dis*. 2003. doi: 10.1086/374002.

16. Barton ES, White DW, Cathelyn JS, Brett-McClellan KA, Engle M, Diamond MS, Miller VL, Virgin HW. Herpesvirus Latency Confers Symbiotic Protection from Bacterial Infection. *Nature*. 2007;447:326-9. doi: 10.1038/nature05762.

17. Smithey MJ, Venturi V, Davenport MP, Buntzman AS, Vincent BG, Frelinger JA, Nikolich-Žugich J. Lifelong CMV Infection Improves Immune Defense in Old Mice by Broadening the Mobilized TCR Repertoire against Third-Party Infection. *Proc Natl Acad Sci U S A*. 2018;115:E6817-e25. doi: 10.1073/pnas.1719451115.

18. Sandalova E, Laccabue D, Boni C, Tan AT, Fink K, Ooi EE, Chua R, Shafaeddin Schreve B, Ferrari C, Bertoletti A. Contribution of Herpesvirus Specific CD8 T Cells to Anti-Viral T Cell Response in Humans. *PLoS Pathog*. 2010;6:e1001051. doi: 10.1371/journal.ppat.1001051.

19. Sharma S, Thomas PG. The Two Faces of Heterologous Immunity: Protection or Immunopathology. *J Leukoc Biol*. 2014;95:405-16. doi: 10.1189/jlb.0713386.

20. Stern M, Hadaya K, Hönger G, Martin PY, Steiger J, Hess C, Villard J. Telomeric rather than centromeric activating KIR genes protect from cytomegalovirus infection after kidney transplantation. *Am J Transplant*. 2011;11(6):1302-1307. doi: 10.1111/j.1600-6143.2011.03516.x.

21. Currenti J, Chopra A, John M, Leary S, McKinnon E, Alves E, Pilkinton M, Smith R, Barnett L, McDonnell WJ, Lucas M, Noel F, Mallal S, Conrad JA, Kalams SA, Gaudieri S. Deep Sequence Analysis of HIV Adaptation Following Vertical Transmission Reveals the Impact of Immune Pressure on the Evolution of HIV. *PLoS Pathog*. 2019;15:e1008177. doi: 10.1371/journal.ppat.1008177.

22. Kijak GH. Lost in Translation: Implications of HIV-1 Codon Usage for Immune Escape and Drug Resistance. *AIDS Rev*. 2004;6:54-60.

23. Du VY, Bansal A, Carlson J, Salazar-Gonzalez JF, Salazar MG, Ladell K, Gras S, Josephs TM, Heath SL, Price DA, Rossjohn J, Hunter E, Goepfert PA. HIV-1-Specific CD8 T Cells Exhibit Limited Cross-Reactivity during Acute Infection. *J Immunol*. 2016;196(8):3276-3286. doi: 10.4049/jimmunol.1502411.

24. Almeida C-AM, Bronke C, Roberts SG, McKinnon E, Keane NM, Chopra A, Kadie C, Carlson J, Haas DW, Riddler SA, Haubrich R, Heckerman D, Mallal S, John M. Translation of HLA-HIV Associations to the Cellular Level: HIV Adapts to Inflate CD8 T

Cell Responses against Nef and HLA-Adapted Variant Epitopes. *J Immunol*. 2011;187:2502-13. doi: 10.4049/jimmunol.1100691.

25. Brumme ZL, Brumme CJ, Carlson J, Streeck H, John M, Eichbaum Q, Block BL, Baker B, Kadie C, Markowitz M, Jessen H, Kelleher AD, Rosenberg E, Kaldor J, Yuki Y, Carrington M, Allen TM, Mallal S, Altfeld M, Heckerman D, Walker BD. Marked Epitope- and Allele-Specific Differences in Rates of Mutation in Human Immunodeficiency Type 1 (HIV-1) Gag, Pol, and Nef Cytotoxic T-Lymphocyte Epitopes in Acute/Early HIV-1 Infection. *J Virol*. 2008;82:9216-27. doi: 10.1128/JVI.01041-08.

26. Currenti J, Law BMP, Qin K, John M, Pilkinton MA, Bansal A, Leary S, Ram R, Chopra A, Gangula R, Yue L, Warren C, Barnett L, Alves E, McDonnell WJ, Sooda A, Heath SL, Mallal S, Goepfert P, Kalams SA, Gaudieri S. Cross-Reactivity to Mutated Viral Immune Targets Can Influence CD8(+) T Cell Functionality: An Alternative Viral Adaptation Strategy. *Front Immunol*. 2021;12:746986. doi: 10.3389/fimmu.2021.746986.

27. Mallal S, Asiaee A, Phillips E. Keystone Epitope Theory in Deep Time: How Viral Endogenization and Sexual Dimorphism Wrote the Genomic Syllabus of Immunity. *Zenodo*. 2026. doi: 10.5281/zenodo.18520425.

28. Moore CB, John M, James IR, Christiansen FT, Witt CS, Mallal SA. Evidence of HIV-1 Adaptation to HLA-Restricted Immune Responses at a Population Level. *Science*. 2002;296:1439-43. doi: 10.1126/science.1069660.

29. Bronke C, Almeida C-AM, McKinnon E, Roberts SG, Keane NM, Chopra A, Carlson JM, Heckerman D, Mallal S, John M. HIV Escape Mutations Occur Preferentially at HLA-Binding Sites of CD8 T-Cell Epitopes. *AIDS*. 2013;27:899-905. doi: 10.1097/qad.0b013e32835e1616.

30. Keane NM, Roberts SG, Almeida CAM, Krishnan T, Chopra A, Demaine E, Laird R, Tschochner M, Carlson JM, Mallal S, Heckerman D, James I, John M. High-Avidity, High-IFN-gamma-Producing CD8 T-Cell Responses Following Immune Selection during HIV-1 Infection. *Immunol Cell Biol*. 2012;90:224-34. doi: 10.1038/icb.2011.34.

31. Chikata T, Carlson JM, Tamura Y, Borghan MA, Naruto T, Hashimoto M, Murakoshi H, Le AQ, Mallal S, John M, Gatanaga H, Oka S, Brumme ZL, Takiguchi M. Host-Specific Adaptation of HIV-1 Subtype B in the Japanese Population. *J Virol*. 2014;88:4764-75. doi: 10.1128/JVI.00147-14.

32. Carlson JM, Du VY, Pfeifer N, Bansal A, Tan VYF, Power K, Brumme CJ, Kreimer A, DeZiel CE, Fusi N, Schaefer M, Brockman MA, Gilmour J, Price MA, Kilembe W, Haubrich R, John M, Mallal S, Shapiro R, Frater J, Harrigan PR, Ndung'u T, Allen S, Heckerman D, Sidney J, Allen TM, Goulder PJR, Brumme ZL, Hunter E, Goepfert PA. Impact of Pre-Adapted HIV Transmission. *Nat Med*. 2016;22:606-13. doi: 10.1038/nm.4100.

33. Avila-Rios S, Ormsby CE, Carlson JM, Valenzuela-Ponce H, Blanco-Heredia J, Garrido-

Rodriguez D, Garcia-Morales C, Heckerman D, Brumme ZL, Mallal S, John M, Espinosa E, Reyes-Teran G. Unique Features of HLA-Mediated HIV Evolution in a Mexican Cohort: A Comparative Study. *Retrovirology*. 2009;6:72. doi: 10.1186/1742-4690-6-72.

34. Al-kaabi M, Deshpande P, Firth M, Pavlos R, Chopra A, Basiri H, Currenti J, Alves E, Kalams S, Fellay J, Phillips E, Mallal S, John M, Gaudieri S. Epistatic Interaction between ERAP2 and HLA Modulates HIV-1 Adaptation and Disease Outcome in an Australian Population. *PLoS Pathog*. 2024;20:e1012359. doi: 10.1371/journal.ppat.1012359.

35. Gatanaga H, Brumme ZL, Adland E, Reyes-Terán G, Avila-Rios S, Mejía-Villatoro CR, Hayashida T, Chikata T, Van Tran G, Van Nguyen K, Meza RI, Palou EY, Valenzuela-Ponce H, Pascale JM, Porras-Cortés G, Manzanero M, Lee GQ, Martin JN, Carrington MN, John M, Mallal S, Poon AFY, Goulder P, Takiguchi M, Oka S. Potential for Immune-Driven Viral Polymorphisms to Compromise Antiretroviral-Based Preexposure Prophylaxis for Prevention of HIV-1 Infection. *AIDS*. 2017;31:1935-43. doi: 10.1097/QAD.0000000000001575.

36. John M, Moore CB, James IR, Mallal SA. Interactive Selective Pressures of HLA-Restricted Immune Responses and Antiretroviral Drugs on HIV-1. *Antivir Ther*. 2005;10:551-5.

37. Files JK, Sterrett S, Henostroza S, Fucile C, Maroney K, Fram T, Mallal S, Kalams S, Carlson J, Rosenberg A, Erdmann N, Bansal A, Goepfert PA. HLA-II-Associated HIV-1 Adaptation Decreases CD4(+) T-Cell Responses in HIV-1 Vaccine Recipients. *J Virol*. 2022;96:e0119122. doi: 10.1128/jvi.01191-22.

38. Greenspan NS. Design Challenges for HIV-1 Vaccines Based on Humoral Immunity. *Front Immunol*. 2014;5:335. doi: 10.3389/fimmu.2014.00335.

39. Alves E, Al-Kaabi M, Keane NM, Leary S, Almeida C-AM, Deshpande P, Currenti J, Chopra A, Smith R, Castley A, Mallal S, Kalams SA, Gaudieri S, John M. Adaptation to HLA-Associated Immune Pressure over the Course of HIV Infection and in Circulating HIV-1 Strains. *PLoS Pathog*. 2022;18:e1010965. doi: 10.1371/journal.ppat.1010965.

40. Kawashima Y, Pfafferott K, Frater J, Matthews P, Payne R, Addo M, Gatanaga H, Fujiwara M, Hachiya A, Koizumi H, Kuse N, Oka S, Duda A, Prendergast A, Crawford H, Leslie A, Brumme Z, Brumme C, Allen T, Brander C, Kaslow R, Tang J, Hunter E, Allen S, Mulenga J, Branch S, Roach T, John M, Mallal S, Ogwu A, Shapiro R, Prado JG, Fidler S, Weber J, Pybus OG, Klenerman P, Ndung'u T, Phillips R, Heckerman D, Harrigan PR, Walker BD, Takiguchi M, Goulder P. Adaptation of HIV-1 to Human Leukocyte Antigen Class I. *Nature*. 2009;458:641-5. doi: 10.1038/nature07746.

41. Brockman MA, Chopera DR, Olvera A, Brumme CJ, Sela J, Markle TJ, Martin E, Carlson JM, Le AQ, McGovern R, Cheung PK, Kelleher AD, Jessen H, Markowitz M, Rosenberg E, Frahm N, Sanchez J, Mallal S, John M, Harrigan PR, Heckerman D, Brander C, Walker BD, Brumme ZL. Uncommon Pathways of Immune Escape Attenuate HIV-1 Integrase Replication Capacity. *J Virol*. 2012;86:6913-23. doi: 10.1128/JVI.07133-11.

42. Hertz T, Nolan D, James I, John M, Gaudieri S, Phillips E, Huang JC, Riadi G, Mallal S, Jojic N. Mapping the Landscape of Host-Pathogen Coevolution: HLA Class I Binding and Its Relationship with Evolutionary Conservation in Human and Viral Proteins. *J Virol*. 2011;85:1310-21. doi: 10.1128/JVI.01966-10.

43. Kiepiela P, Leslie AJ, Honeyborne I, Ramduth D, Thobakgale C, Chetty S, Rathnavalu P, Moore C, Pfafferott KJ, Hilton L, Zimbwa P, Moore S, Allen T, Brander C, Addo MM, Altfeld M, James I, Mallal S, Bunce M, Barber LD, Szinger J, Day C, Klenerman P, Mullins J, Korber B, Coovadia HM, Walker BD, Goulder PJR. Dominant Influence of HLA-B in Mediating the Potential Co-Evolution of HIV and HLA. *Nature*. 2004;432:769-75. doi: 10.1038/nature03113.

44. Rolland M, Manocheewa S, Swain JV, Lanxon-Cookson EC, Kim M, Westfall DH, Larsen BB, Gilbert PB, Mullins JI. HIV-1 Conserved-Element Vaccines: Relationship between Sequence Conservation and Replicative Capacity. *J Virol*. 2013;87:5461-7. doi: 10.1128/jvi.03033-12.

45. Ondondo B, Murakoshi H, Clutton G, Abdul-Jawad S, Wee EGT, Gatanaga H, Oka S, McMichael AJ, Takiguchi M, Korber B, Hanke T. Novel Conserved-Region T-Cell Mosaic Vaccine with High Global HIV-1 Coverage Is Recognized by Protective Responses in Untreated Infection. *Mol Ther*. 2016;24:832-42. doi: 10.1038/mt.2016.3.

46. Mateus J, Grifoni A, Tarke A, Sidney J, Ramirez SI, Dan JM, Burger ZC, Rawlings SA, Smith DM, Phillips E, Mallal S, Lammers M, Rubiro P, Quiambao L, Sutherland A, Yu ED, da Silva Antunes R, Greenbaum J, Frazier A, Markmann AJ, Premkumar L, de Silva A, Peters B, Crotty S, Sette A, Weiskopf D. Selective and Cross-Reactive SARS-CoV-2 T Cell Epitopes in Unexposed Humans. *Science*. 2020;370:89-94. doi: 10.1126/science.abd3871.

47. Tarke A, Sidney J, Kidd CK, Dan JM, Ramirez SI, Yu ED, Mateus J, da Silva Antunes R, Moore E, Rubiro P, Methot N, Phillips E, Mallal S, Frazier A, Rawlings SA, Greenbaum JA, Peters B, Smith DM, Crotty S, Weiskopf D, Grifoni A, Sette A. Comprehensive Analysis of T Cell Immunodominance and Immunoprevalence of SARS-CoV-2 Epitopes in COVID-19 Cases. *Cell Rep Med*. 2021;2:100204. doi: 10.1016/j.xcrm.2021.100204.

48. Tarke A, Zhang Y, Methot N, Narowski TM, Phillips E, Mallal S, Frazier A, Filaci G, Weiskopf D, Dan JM, Premkumar L, Scheuermann RH, Sette A, Grifoni A. Targets and Cross-Reactivity of Human T-Cell Recognition of Common Cold Coronaviruses. *Cell Rep Med*. 2023;4:101088. doi: 10.1016/j.xcrm.2023.101088.

49. Leary S, Gaudieri S, Parker M, Chopra A, James I, Pakala S, Alves E, John M, Lindsey B, Keeley A, Rowland-Jones S, Swanson M, Ostrov D, Bubenik J, Das S, Sidney J, Sette A, consortium C-GU, De Silva T, Phillips E, Mallal S. Generation of a Novel SARS-CoV-2 Sub-Genomic RNA Due to the R203K/G204R Variant in Nucleocapsid: Homologous Recombination Has Potential to Change SARS-CoV-2 at Both Protein and RNA Level. *Pathog Immun*. 2021;6:27-49. doi: 10.20411/pai.v6i2.460.

50. Maguire C, Chen J, Rouphael N, Pickering H, Phan HV, Glascock A, Chu V, Dandekar R, Corry D, Kheradmand F, Baden LR, Selaky R, McComsey GA, Haddad EK, Cairns CB, Pulendran B, Sesma AF, Simon V, Metcalf JP, Agudelo Higuita NI, Messer WB, David MM, Nadeau KC, Kraft M, Bime C, Schaenman J, Erle D, Calfee CS, Atkinson MA, Brackenridge SC, Ehrlich LIR, Montgomery RR, Shaw AC, Hough CL, Geng LN, Hafler DA, Augustine AD, Becker PM, Peters B, Ozonoff A, Kim-Schulze SH, Krammer F, Bosinger S, Eckalbar W, Altman MC, Wilson M, Guan L, Kleinstein SH, Network I, Smolen KK, Reed EF, Levy O, Maecker H, Hunt P, Steen H, Diray-Arce J, Langelier CR, Melamed E. Chronic Viral Reactivation and Associated Host Immune Response and Clinical Outcomes in Acute COVID-19 and Post-Acute Sequelae of COVID-19. *bioRxiv*. 2024. doi: 10.1101/2024.11.14.622799.

51. Woldemeskel BA, Garliss CC, Blankson JN. SARS-CoV-2 mRNA Vaccines Induce Broad CD4+ T Cell Responses That Recognize SARS-CoV-2 Variants and HCoV-NL63. *The J Clin Invest*. 2021;131. doi: 10.1172/JCI149335.

52. Pothast CR, Dijkland RC, Thaler M, Hagedoorn RS, Kester MGD, Wouters AK, Hiemstra PS, van Hemert MJ, Gras S, Falkenburg JHF, Heemskerk MHM. SARS-CoV-2-Specific CD4+ and CD8+ T Cell Responses Can Originate from Cross-Reactive CMV-Specific T Cells. *eLife*. 2022;11:e82050. doi: 10.7554/eLife.82050.

53. Malouli D, Hansen SG, Nakayasu ES, Marshall EE, Hughes CM, Ventura AB, Gilbride RM, Lewis MS, Xu G, Kreklywich C, Whizin N, Fischer M, Legasse AW, Viswanathan K, Siess D, Camp DG, Axthelm MK, Kahl C, DeFilippis VR, Smith RD, Streblow DN, Picker LJ, Früh K. Cytomegalovirus pp65 Limits Dissemination but Is Dispensable for Persistence. *The Journal of clinical investigation*. 2014;124:1928-44. doi: 10.1172/JCI67420.

54. Lineburg KE, Grant EJ, Swaminathan S, Chatzileontiadou DSM, Szeto C, Sloane H, Panikkar A, Raju J, Crooks P, Rehan S, Nguyen AT, Lekieffre L, Neller MA, Tong ZWM, Jayasinghe D, Chew KY, Lobos CA, Halim H, Burrows JM, Riboldi-Tunnicliffe A, Chen W, D'Orsogna L, Khanna R, Short KR, Smith C, Gras S. CD8+ T Cells Specific for an Immunodominant SARS-CoV-2 Nucleocapsid Epitope Cross-React with Selective Seasonal Coronaviruses. *Immunity*. 2021;54:1055-65. doi: 10.1016/j.immuni.2021.04.006.

55. Nguyen THO, Rowntree LC, Petersen J, Chua BY, Hensen L, Kedzierski L, van de Sandt CE, Chaurasia P, Tan H-X, Habel JR, Zhang W, Allen LF, Earnest L, Mak KY, Juno JA, Wragg K, Mordant FL, Amanat F, Krammer F, Mifsud NA, Doolan DL, Flanagan KL, Sonda S, Kaur J, Wakim LM, Westall GP, James F, Mouhtouris E, Gordon CL, Holmes NE, Smibert OC, Trubiano JA, Cheng AC, Harcourt P, Clifton P, Crawford JC, Thomas PG, Wheatley AK, Kent SJ, Rossjohn J, Torresi J, Kedzierska K. CD8(+) T Cells Specific for an Immunodominant SARS-CoV-2 Nucleocapsid Epitope Display High Naive Precursor Frequency and TCR Promiscuity. *Immunity*. 2021;54:1066-82. doi: 10.1016/j.immuni.2021.04.009.

56. Le Bert N, Tan AT, Kunasegaran K, Tham CYL, Hafezi M, Chia A, Chng MHY, Lin M,

Tan N, Linster M, Chia WN, Chen MIC, Wang L-F, Ooi EE, Kalimuddin S, Tambyah PA, Low JG-H, Tan Y-J, Bertoletti A. SARS-CoV-2-Specific T Cell Immunity in Cases of COVID-19 and SARS, and Uninfected Controls. *Nature*. 2020;584:457-62. doi: 10.1038/s41586-020-2550-z.

57. Notarbartolo S. T-Cell Immune Responses to SARS-CoV-2 Infection and Vaccination. *Vaccines*. 2024;12:1126. doi: 10.3390/vaccines12101126.

58. Reese TA, Wakeman BS, Choi HS, Hufford MM, Huang SC, Zhang X, Buck MD, Jezewski A, Kambal A, Liu CY, Goel G, Murray PJ, Xavier RJ, Kaplan MH, Renne R, Speck SH, Artyomov MN, Pearce EJ, Virgin HW. Helminth Infection Reactivates Latent γ-Herpesvirus Via Cytokine Competition at a Viral Promoter. *Science*. 2014;345:573-7. doi: 10.1126/science.1254517.

59. Daud II, Ogolla S, Amolo AS, Namuyenga E, Simbiri K, Bukusi EA, Ng'ang'a ZW, Ploutz-Snyder R, Sumba PO, Dent A, Rochford R. Plasmodium Falciparum Infection Is Associated with Epstein-Barr Virus Reactivation in Pregnant Women Living in Malaria Holoendemic Area of Western Kenya. *Matern Child Health J*. 2015;19:606-14. doi: 10.1007/s10995-014-1546-4.

60. Gordon CL, Lee LN, Swadling L, Hutchings C, Zinser M, Highton AJ, Capone S, Folgori A, Barnes E, Klenerman P. Induction and Maintenance of CX3CR1-Intermediate Peripheral Memory CD8(+) T Cells by Persistent Viruses and Vaccines. *Cell Rep*. 2018;23:768-82. doi: 10.1016/j.celrep.2018.03.074.

61. Matthews PC, Prendergast A, Leslie A, Crawford H, Payne R, Rousseau C, Rolland M, Honeyborne I, Carlson J, Kadie C, Brander C, Bishop K, Mlotshwa N, Mullins JI, Coovadia H, Ndung'u T, Walker BD, Heckerman D, Goulder PJR. Central Role of Reverting Mutations in HLA Associations with Human Immunodeficiency Virus Set Point. *J Virol*. 2008;82:8548-59. doi: 10.1128/JVI.00580-08.

62. Salomé B, Sfakianos JP, Ranti D, Daza J, Bieber C, Charap A, Hammer C, Banchereau R, Farkas AM, Ruan DF, Izadmehr S, Geanon D, Kelly G, de Real RM, Lee B, Beaumont KG, Shroff S, Wang YA, Wang Y-c, Thin TH, Garcia-Barros M, Hegewisch-Solloa E, Mace EM, Wang L, O'Donnell T, Chowell D, Fernandez-Rodriguez R, Skobe M, Taylor N, Kim-Schulze S, Sebra RP, Palmer D, Clancy-Thompson E, Hammond S, Kamphorst AO, Malmberg K-J, Marcenaro E, Romero P, Brody R, Viard M, Yuki Y, Martin M, Carrington M, Mehrazin R, Wiklund P, Mellman I, Mariathasan S, Zhu J, Galsky MD, Bhardwaj N, Horowitz A. NKG2A and HLA-E Define an Alternative Immune Checkpoint Axis in Bladder Cancer. *Cancer Cell*. 2022;40:1027-43. doi: 10.1016/j.ccell.2022.08.005.

63. Martin MP, Gao X, Lee JH, Nelson GW, Detels R, Goedert JJ, Buchbinder S, Hoots K, Vlahov D, Trowsdale J, Wilson M, O'Brien SJ, Carrington M. Epistatic interaction between KIR3DS1 and HLA-B delays the progression to AIDS. *Nat Genet*. 2002;31(4):429-434. doi: 10.1038/ng934.
